# Range shifts of overwintering birds depend on habitat type, snow conditions and habitat specialization

**DOI:** 10.1007/s00442-022-05209-5

**Published:** 2022-06-29

**Authors:** Laura Bosco, Yanjie Xu, Purabi Deshpande, Aleksi Lehikoinen

**Affiliations:** 1 The Helsinki Lab of Ornithology, Finnish Museum of Natural History, University of Helsinki, Helsinki, Finland; 2 Research Programme in Organismal and Evolutionary Biology, Faculty of Biological and Environmental Sciences, University of Helsinki, 00011 Helsinki, Finland

**Keywords:** Distribution shifts, Global warming, Habitat use, Bird monitoring, Climate niche

## Abstract

Climatic warming is forcing species to shift their ranges poleward, which has been demonstrated for many taxa globally. Yet, the influence of habitat types on within- and among-species variations of distribution shifts has rarely been studied, especially during the non-breeding season. Here, we investigated habitat-specific shift distances of northern range margins and directions of the distribution center based on long-term data of overwintering birds in Finland. Specifically, we explored influences of habitat type, species’ snow depth tolerance, species’ climatic niche and habitat specialization on range shifts during the past 40 years in 81 bird species. Birds overwintering in arable land shifted more clearly toward north compared to birds of the same species in rural and forest habitats, while the northern range margin shift distances did not significantly differ among the habitat types. Range shifts were more linked with the species’ snow depth tolerance rather than species’ climatic niche. Snow depth tolerance of species was negatively associated with the eastward shift direction across all habitats, while we found habitat-specific patterns with snow depth for northward shift directions and northern margin shift distances. Species with stronger habitat specializations shifted more strongly toward north as compared to generalist species, whereas the climatic niche of bird species only marginally correlated with range shifts, so that cold-dwelling species shifted longer distances and more clearly eastward. Our study reveals habitat-specific patterns linked to snow conditions for overwintering boreal birds and highlights the importance of habitat availability and preference in climate driven range shifts.

**Supplementary Information:**

The online version contains supplementary material available at 10.1007/s00442-022-05209-5.

## Introduction

Anthropogenic climate change is widely recognized as a major threat to global biodiversity (Thomas and Williamson 2012), exacerbating the sixth mass extinction in the history of our planet (Bellard et al. 2012). High-latitude and high-elevation ecosystems are at risk of severe impacts globally, while arctic ecosystems are particularly vulnerable (Masson-Delmotte et al. 2018). It is thus crucial to understand how communities respond and adapt to the spatiotemporal variability in their environment, making it a key research area in ecology (Begon et al. 1986). To design effective conservation and management strategies and mitigation measures to climate change, we need a holistic understanding of the ecological, physiological, genetic, and biogeographical mechanisms driving species responses to climate change (Bonebrake et al. 2018). Ongoing responses to climate change are already visible among many taxa, where a growing body of research has shown that species respond to climate-driven changes in their climatic niche in multiple ways (Both et al. 2006; Chen et al. 2011; Bellard et al. 2012; Stephens et al. 2016; Ryding et al. 2021).

Changes in the distribution of species linked with climatic conditions are well researched. In the Northern Hemisphere, a clear consensus exists that in general species are shifting poleward (Devictor et al. 2008; Mason et al. 2015; Lehikoinen and Virkkala 2016); however, the velocity of these shifts differs between different taxa (e.g., butterflies shift faster than birds, Devictor et al. 2012). There are also variations in poleward shifts between different biogeographical populations of conspecifics (Lehikoinen et al. 2021a, b) and speed of shifts can be facilitated by increased habitat quality including protected areas (Lehikoinen et al. 2019). Moreover, species responses to climate change are modified through their habitat use. For instance, species-specific variation in poleward shifts of invertebrates in the UK can be explained by habitat availability (Platts et al. 2019), and the rapid northward expansion of wintering birds in the UK is linked to resource availability related to human-modified landscapes (Van Doren et al. 2021). In general, anthropogenic modifications of the landscape and resource availability can drastically influence the distribution and migratory behavior of species, through e.g., supplementary feeding (Greig et al. 2017). Also, in the case of Finnish butterflies, habitat availability plays a crucial role in whether a species can expand its range poleward or not (Pöyry et al. 2009). Other research revealed that odonates specialized to lentic habitats have shifted their home ranges poleward faster than those adapted to lotic habitats (Grewe et al. 2013), or that bird species breeding in scrub and grassland habitats show substantial poleward shifts, while urban, wetland and woodland-breeding species show a non-significant poleward shift (Hovick et al. 2016). It was proposed before that species habitat specialization is linked to its dispersal ability, which in turn influences a species potential to shift its range. In Jocque et al. (2010), the authors suggest a trade-off between dispersal ability and ecological specialization, so that species with a higher habitat specialization would have lower dispersal rates, whereas the opposite pattern has been shown in an observational study on birds (Martin and Fahrig 2018).

Range shifts can be influenced by changes in climatic conditions during both breeding and wintering seasons. Winter conditions vary geographically more than summer conditions (Bonan et al. 2003) and hence it is crucial to understand how species are moving poleward in response to winter conditions rather than during the warmer season solely (Lehikoinen et al. 2021a, b). Overwintering is a key component of the biology of organisms that live in temperate, polar, and alpine habitats, and has driven the evolution of extreme phenotypes such as dormancy and migration (Williams et al. 2015). The primary abiotic drivers of the biological impacts of winter conditions on terrestrial systems are temperature and snow cover (Williams et al. 2015), which are often highly correlated (Deshpande et al. 2022). As a result of the variables being correlated and snow cover data relevant for animal populations being difficult to collect, studies often chose to explore the effect of temperature over snow cover (Boelman et al. 2019). Yet, the interaction between habitat use and varying snow or temperature conditions on range shifts of species have only weakly been investigated so far (but see Deshpande et al. 2022). In Finland, reduced snow depth resulting from climate change varies widely across regions making it a complex system for animals to navigate while overwintering (Luomaranta et al. 2019). Climatic conditions during winter can influence habitat use and subsequently affect distribution changes in both aquatic and terrestrial ecosystems. For instance, in open arable land the decrease in snow cover (due to climate change) can significantly increase the accessibility of food resources (Henderson et al. 2004; Goławski and Kasprzykowski 2010). Diving ducks, which are linked to open water areas, have shifted their wintering sites more rapidly than other waterbird species, occurring in coastal areas or farmlands (Pavón-Jordán et al. 2019). Land birds on the other hand can be attracted to human settlements, where access to food is easier during harsh winter conditions (Goławski and Kasprzykowski 2010), whereas in milder conditions birds are more evenly distributed or even aggregated in rural areas (Deshpande et al. 2022). In addition to habitat availability, the climatic niche of a species, or entire community, has been shown to be linked to climate change sensitivity (Thuiller et al. 2005). This climatic niche can be quantified by simple measures such as the Species Temperature Index (STI), where lower STI values represent cold-dwelling and higher STI values warm-dwelling species (Devictor et al. 2008; Santangeli and Lehikoinen 2017). Previous studies have found that cold-dwelling species shifted faster poleward than warm-dwelling species during the breeding season (Virkkala and Lehikoinen 2014; Tayleur et al. 2016; Lehikoinen et al. 2021a, b).

In this study, we aimed to understand how habitat type influences intra-specific range shifts of species during the winter season, measured as changes in their central gravity of abundance and northern range margin over time. Using long-term winter bird monitoring data covering 81 species from Finland, we sought to answer the following questions: (i) do abundances and range margins of species shift differently dependent on the habitat type they overwinter in, (ii) is species’ tolerance to snow depth influencing the different abundance and range margin shifts per habitat type, (iii) is this difference influenced by their overwintering thermal preference (measured by STI), and (iv) do species shift farther in their preferred (main) habitat compared to other (sub) habitats and is the distance and direction of the shift dependent on how specialized the species is on the main habitat.

## Methods

### Winter bird counts and data selection

We used a long-term dataset of bird observations based on standardized winter bird counts from Finland starting in the early 1950s (Koskimies and Väisänen 1991). Since the 1970s, the counts have consisted of a three-visit survey between November and March, wherein volunteers count all birds encountered on a transect band (of on average 10 km length). In each survey, trained observers walk along the predefined route and count all birds detected visually or auditorily. From 1987, volunteers have also recorded the length of each type of habitat along the transect, i.e., the actual distance walked through a certain habitat, as one of eight habitat types: (i) garbage dumping ground or fur farm, (ii) urban settlement, (iii) rural settlement, (iv) arable land and pasture, (v) forest, (vi) clear-cut area or stand of saplings, (vii) reed bed or shore scrub and (viii) ‘other’, which includes, for example, birds in wetlands or on active migration. Usually, volunteers responsible for a transect carry out the transect over multiple years (up to decades) and record the change in biotopes over the years in their transect. Transects are handed over to the new observers under the supervision of the previous volunteers. The census scheme encourages volunteers to include multiple biotopes in the routes they census, so that the route would represent the habitats in the same proportion as they appeared in the surrounding area. Due to the conditions in the winter, i.e., shortage of daylight hours, birds are foraging actively and visibly but not moving larger distances due to the cold weather. We assume that the detectability of birds was similarly high within habitats across years, but possibly varied across habitats (Deshpande et al. 2022). A large part of the routes has been active for decades what increases the reliability of comparison between study periods (Fraixedas et al. 2015; Deshpande et al. 2022).

Among a total of > 76,000 winter bird surveys from > 4100 transects, we restricted our analyses to two time periods, with period one including the years 1987–2000 and period two 2010–2020 (hereafter period 1 and 2, respectively), where the habitat data had been collected from the transects. We further only selected the mid-winter counts between 25 December and 7 January, as this period had the highest number of surveys (see Lehikoinen et al. 2013; Fraixedas et al. 2015). Next, we excluded surveys where information on the habitat type was missing, so that our final dataset had information on the habitat type per transect length and bird observations. We summarized the original eight habitat classifications to four classes for the purpose of this study (numbers in brackets refer to the original classes, see above): urban (i + ii), rural settlements (iii), arable land (iv), and forest (v + vi + vii). Note that the habitat classification of ‘reed’ includes reed beds and shore scrubs, which most often include trees at the ends of the shores. Reed beds are a rather uncommon habitat type in Finland and several forest species are using this habitat also during winter, which is why we included it in the habitat type ‘forest’. Observations from the class “other” (viii) were dropped from our dataset. The sampling of different habitats has remained relatively stable since the 1980s. The most important occurred changes have been reduced amount of dumping grounds (due to environmental regulations), which are included to the urban areas, and moderate increase in area of clear cuts and stand of saplings, which mimic the increased logging intensity (included to ‘forest’, Lehikoinen and Väisänen 2014; National Resources Institute Finland 2022). The final data consisted of a total of 63,249 km of transects during period 1 (ESM Fig. S1; 13,680 km, 13,027 km, 9706 km, and 26,837 km in urban, rural settlements, arable land, and forest habitats, respectively), while 57′503 km of transects were surveyed during period 2 (ESM Fig. S1; 15,934 km, 10,778 km, 7086 km, and 23,704 km in urban, rural settlements, arable land, and forest habitats, respectively). We generated 100 × 100 km grids covering the extent of Finland and assigned each transect to the grid that contained the geometric center of the transect (ESM Fig. S1). We calculated the habitat-specific sum of transect lengths in each grid per period and included those grids with a total of > 1 km of transects surveyed per habitat type in the subsequent analysis. We explored different minimal survey length thresholds (see ESM Fig. S14) with no substantial differences among the thresholds, so we used a 1-km threshold allowing us to include more observations. To reduce the potential bias from counting rare species, we only included species with ≥ 10 individuals observed per habitat type and period and ≥ 40 individuals observed per habitat type in both periods together, which resulted in a total of 81 bird species that were included.

### Species range shift

We calculated northern range margins by averaging the latitudes of the three northernmost grids per habitat, period, and species. Thus, shifted distances in northern range margins were quantified by their differences between the two periods, whereby we excluded those species-habitat observations where the habitat-specific northern margin was farther north than 66.85° N. Hence, only observations where the species had space to shift northward were included in this analysis (i.e., 4 species and 54 observations were excluded, resulting in 77 species in the dataset). A total of 180 observations were included in subsequent modeling, each of which was one species and habitat type combination (i.e., where the species were observed, as not all species occurred in all four habitat types; see ESM Table S5). We did not include southern range margin shifts since very few species in the dataset would have their trailing edge within Finland, which was reflected in no detectable shift in the southern margin in our data (ESM Fig. S4b).

The species relative densities (*n*/km) (hereafter densities) were calculated separately for each 100 km grid per habitat and period, by dividing total abundances of each species per habitat type by the total surveyed transect length (km). We used these grid-specific densities to calculate arithmetic central gravity of densities for each species per habitat and period. This was performed by first calculating the latitude using mean densities per each latitude grid row and then calculating the longitude using mean densities per each grid column (Virkkala and Lehikoinen 2014). Since the central gravity of densities was affected by the location of transects inside each grid, the mean of coordinates of all transects per grid per period was used, instead of the grids’ geographic centroids (after Lehikoinen and Virkkala 2016). Based on latitude and longitude of the centers of gravity per period, we calculated the distance and geographic direction of the density shift per species between the two periods for each of the four habitat types separately. A total of 234 observations were included in subsequent modeling, each of which was one species and habitat type combination with 41 observations in arable land, 64 in forests, 61 in rural settlements and 68 in urban areas (see ESM Table S5).

### Species habitat specificity

To test whether species shift differently in the main overwintering habitat with highest species-specific densities (main habitat) compared to other used habitats (subhabitat), we used winter bird counts from a period in between our two study periods, i.e., years 2000–2009, as an independent dataset from our study data, but nevertheless representative for the habitat-specific overwintering behavior of Finnish birds. We applied the same habitat classification steps as described above and then calculated species densities per habitat type for the 81 bird species included in our study. The habitat type with the highest absolute density was subsequently classified as ‘main’, while the remaining habitats were classified as ‘sub’ (see also ESM Table S6). We also quantified species habitat specialization as a continuous variable by calculating their habitat-specific densities $(\mathrm{density per habitat} / \mathrm{total density over all habitats})$, and used the habitat-specific density of the main habitat type per species as a specialization proxy in the modeling (see also ESM Table S6).

### Explanatory variables

We downloaded gridded daily snow depth data matching the bird survey time window (between 24 December and 7 January for all years in period one and two) from the Finnish Meteorological Institute (https://etsin.fairdata.fi/dataset/c63d696a-8d42-44aa-8508-9024ee05cfa7), which is reported at a 10 km × 10 km spatial resolution and based on automatic measurements of snow depths (cm) with an ultrasound snow sensor and corrected for, e.g., topography and water bodies by kriging interpolation (more details: Aalto et al. 2016). We chose snow depth as a proxy for species’ ability to tolerate snow conditions, as it has been recently shown that snow is a better predictor for overwintering bird abundances in Finland than temperature, although the two predictors are often highly correlated (Deshpande et al. 2022). We calculated the median snow depth per winter and period and averaged the values to the 100 km grids used for the bird data processing (see “Species range shift” above; ESM Fig. S15 and S16). Since the final bird range shift dataset consisted of unique species *x* habitat combinations (i.e., one observation per species in each habitat type it has been observed), we finally calculated the habitat-specific median snow depth in period two (p2), by only using the 100 × 100 km grids (“please see data description above”) where the species was present in period one in the respective habitat type. For example, if a species was present in ten grids in urban and five grids in forest habitats in period one, we calculated the median snow depth in period two across the ten urban and across the five forest habitat grids. Therefore, species which occur in snow-rich areas and habitats have high snow depth values and species which avoid snow received low values. To model the influence of species’ thermal preferences, we included the winter-specific species temperature index (STI in °C; available at Lehikoinen et al. 2021a, b) of each species present in the dataset.

### Statistical analyses

**Fig. 1:**
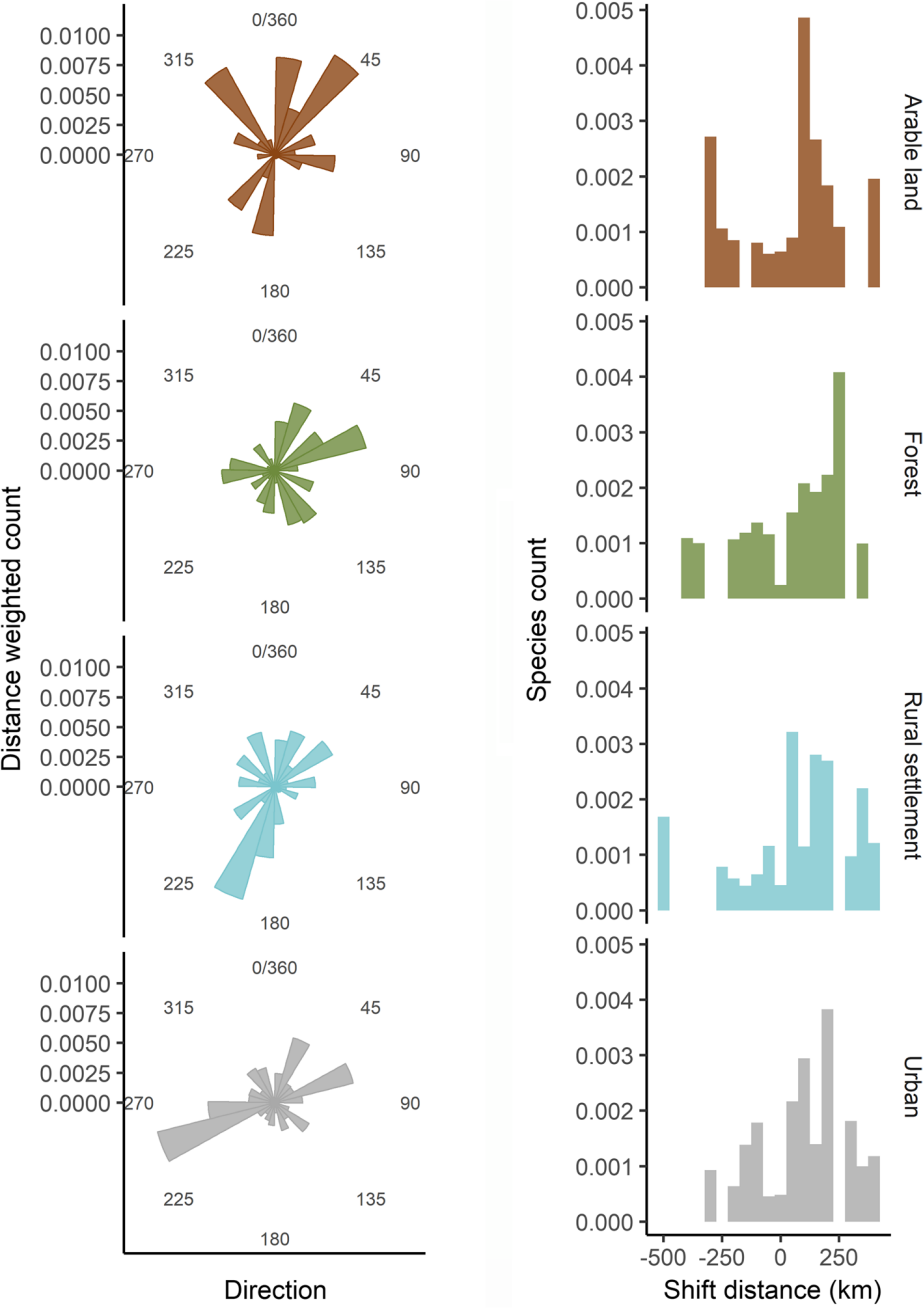
Habitat-specific circular histograms of the range shift direction patterns weighted by distance (left side) and histograms depicting the distance of northern margin shift, where the *y*-axes of histograms show the species counts (right side)

We ran separate analyses for the response variables: (i) shift distance of northern range margins in each habitat and (ii) direction (eastward and northward, see below) of central gravity shifts, while always testing the same set of predictors in all models. For distance of northern range margin shifts, we fitted lmer models and always included the factor species (77 levels) as a random effect to account for varying intercepts among species. To model circular data such as compass direction of range shifts with linear regression models, we transformed the shift direction to a linear expression of northward (shift along the latitudinal axis) and eastward (longitudinal axis) shift (sensu Guyot et al. 2017). To do so, we first transformed directions from degrees to radians $(\mathrm{direction}/360\times 2\times \pi )$ and then calculated the sine and cosine for a measure of eastward (ranging from − 1 = west, to + 1 = east) and northward (− 1 = south, + 1 = north) directions, respectively. Since the assumption of normality of residuals was met (see ESM Fig. S6 and S7), we separately modeled eastward and northward range shifts, by fitting mixed effect linear regression models (‘lmer’, R package ‘lme4’, Bates et al. 2015). We can likely interpret range shifts that happen over longer distances as more directional shifts in species’ central gravities, whereas short(er) shift distances are more error prone and could be attributed to random movement. Thus, we aimed to give more weight to the directionality of longer shifts, by adding the range shift distance of the central gravity as a weight in all eastward and northward direction models to account for the notable variation in distance among the range shifts (Fig. 1). We ran a sensitivity analysis to explore whether effects change when excluding model weights, which was the case for northward shift directions, implying that mainly the longer shifts showed stronger relationships in their directionality along the latitudinal axis with our predictor variables (see ESM Table S4). The factor species was always included as a random intercept term (81 levels).

To answer the first three research questions, we ran the following models for all response variables, and present the best model according to lowest AIC value per response variable, while the others (lower ranked models) are presented in the ESM (Tables S1 and S2).

m1: y ~ habitat,

m2: y ~ habitat + snow p2,

m3: y ~ habitat × snow p2,

m4: y ~ habitat + STI,

m5: y ~ habitat × STI.

To test range shift differences in main vs. subhabitats, we reduced the dataset to those species with occurrences in > 2 habitat types, by dropping 15 species with only one habitat type (66 species left, see ESM Table S6). From there, we tested differences in habitat-specific shifts of northern range margins, and eastward and northward shift directions as a function of main *vs*. subhabitat type classification using linear mixed models (gaussian error distribution) with species as random intercept term.

To test range shift differences for habitat specialization we reduced the dataset to unique species observations, such that per species only the range shift information (i.e., shift distance of northern margin, eastward and northward shift direction) of their main overwintering habitat (habitat = main) was retained (i.e., 80 observations from 80 species, as one species had no main habitat in our dataset: *Plectrophenax nivalis*). We then again modeled northern range margin shift distance, eastward and northward shift directions against the habitat specialization using simple linear regression models (lm).

Finally, linear regressions of model predictions were plotted for variables with significant effects by averaging the other terms in the model, if there were any present (function ‘effect’ in the R package ‘effects’, Fox and Weisberg 2019). For all data processing and statistical analyses, we used R software (R version 4.0.3 2020-10-10, R Core Team 2020).

## Results

Shift distances of northern margins were quite equally distributed along the latitudinal axis, with distances ranging from 0 to 413.75 km (mean ± SD = 111.06 ± 92.09 km) toward north and 0–523.33 km (90.74 ± 100.51 km) toward south (ESM Fig. S3). In general, species were shifting northward in their northern range margins in all four habitat types (i.e., positive latitudinal distance values), but there were no clear differences between the habitat types of rural settlements (mean ± SD: 46 ± 139 km), urban (44 ± 125 km), arable land (37 ± 134 km) and forest (35 ± 147 km).

There was considerable variation in the direction of central gravity shifts among the four habitat types (Fig. 1), where birds mainly shifted northward in arable land (Fig. 1a). Shifts in forest habitats were mainly toward the northeast (Fig. 1c), whereas most distances of these shifts were less than 150 km (Fig. 1d). In contrast, Pm birds in rural settlements and urban habitats showed density shifts toward the west and south directions, respectively (Fig. 1c, d).

### Northern range margin shift distances

The top performing model was the interaction model habitat × snow (m3) (Table 1). Snow depth in period 2 had an overall marginal negative connection to shift distance in the northern range margin meaning that species with lower snow depth preference in period 2 shifted their range margins further north across all habitat types (Table 1, ESM Fig. S8a–d, ESM Fig. S10). However, according to the significant positive interaction between snow depth and habitat, (Table 1), snow depth tolerance of species did not have a major correlation in range margin shifts in rural settlements (ESM Fig. S8c) while there was a clear negative relation in arable land and forest habitats (ESM Fig. S8a, b) and a rather flat one in urban habitats (ESM Fig. S8d). STI was not selected to the top model and thus had no detected connection with northern margin shift distances (Table 1, ESM Table S1).

**Table 1:** Model outputs (estimates, standard errors SE, *t* and *p* values) for the top models of northern (N) range margin distances (m3), and for direction of central gravity shifts, separately for eastward (m2) and northward (m3 and m2) shift directions

Term	Estimate	SE	*t* value	*p* value
*N margin shift distance (m3), N* = *180, AIC* = *646.72, AICw* = *0.519, R*^*2*^ = *0.447*				
**Intercept**	0.183	0.232	0.791	0.430
Habitat forest	0.336	0.277	1.213	0.227
Habitat rural settlement	0.139	0.285	0.486	0.628
Habitat urban	0.363	0.279	1.303	0.194
**Snow period 2**	**-0.518**	**0.271**	**-1.911**	**0.058**
Snow period 2: forest	0.034	0.320	0.105	0.917
**Snow period 2: rural**	**0.620**	**0.307**	**2.019**	**0.045**
Snow period 2: urban	0.249	0.358	0.696	0.487
Random: species (N = 77)	0.907^a^	0.952^a^		
Random: residual	1.278^a^	1.131^a^		
*Eastward shift direction (m2), N* = *234, AIC* = *535.004, AICw* = *0.704, R*^*2*^ = *0.006*				
Intercept	− 0.065	0.103	− 0.626	0.532
Habitat forest	0.179	0.120	1.491	0.137
Habitat rural settlement	0.133	0.121	1.099	0.273
Habitat urban	− 0.003	0.116	0.029	0.977
**Snow period 2**	**−** **0.135**	**0.053**	**−** **2.538**	**0.012**
Random: species (N = 81)	0.154^a^	0.393^a^		
Random: residual	29.385^a^	5.421^a^		
*Northward shift direction (m3), N* = *234, AIC* = *550.240, AICw* = *0.465, R*^*2*^ = *0.008*				
Intercept	0.065	0.167	0.389	0.697
Habitat forest	− 0.066	0.175	− 0.377	0.707
Habitat rural settlement	− 0.073	0.178	− 0.410	0.683
Habitat urban	0.043	0.176	0.245	0.807
Snow period 2	− 0.298	0.216	− 1.384	0.168
**Snow period 2: forest**	**0.442**	**0.222**	**1.994**	**0.047**
**Snow period 2: rural**	**0.482**	**0.221**	**2.184**	**0.030**
Snow period 2: urban	0.268	0.227	1.181	0.239
Random: species (*N* = 81)	0.194^a^	0.440^a^		
Random: residual	29.205^a^	5.404^a^		
*Northward shift direction (m2), N* = *234, AIC* = *550.815, AICw* = *0.349, R*^*2*^ = *0.007*				
**Intercept**	**0.330**	**0.108**	**3.057**	**0.003**
**Habitat forest**	**−** **0.321**	**0.122**	**−** **2.623**	**0.009**
**Habitat rural settlement**	**−** **0.325**	**0.124**	**−** **2.611**	**0.010**
**Habitat urban**	**−** **0.198**	**0.119**	**−** **1.670**	**0.096**
**Snow period 2**	**0.116**	**0.056**	**2.065**	**0.040**
Random: species (*N* = 81)	0.197^a^	0.444^a^		
Random: residual	30.117^a^	5.488^a^		

For range margin shift distances models, we transformed the response variable to units of 100 km to avoid modeling of large values. For each model, the sample size (*N*), AIC, AIC weight (*w*) and conditional *R*^2^ (i.e., the proportion of variance explained by both the fixed and random factors) are given. Significant and marginal effects (*p* < 0.1) are depicted in bold. Continuous predictors were standardized prior to modeling. For the factor habitat, arable land was used as reference level in all models. All other models are shown in ESM Tables S1 and S2

^a^For random effects (species), the variance and standard deviation are shown

### Central gravity shift directions

Eastward shift: The top ranked model explaining eastward shifts in the center of gravity included snow cover depth as a main explanatory variable (m2: habitat + snow p2, Table 1). Species preferring lower snow depth tended to move eastward across all habitats, with no apparent differences among the habitat types (Table 1, ESM Table S2, Fig. S10).

**Fig. 2:**
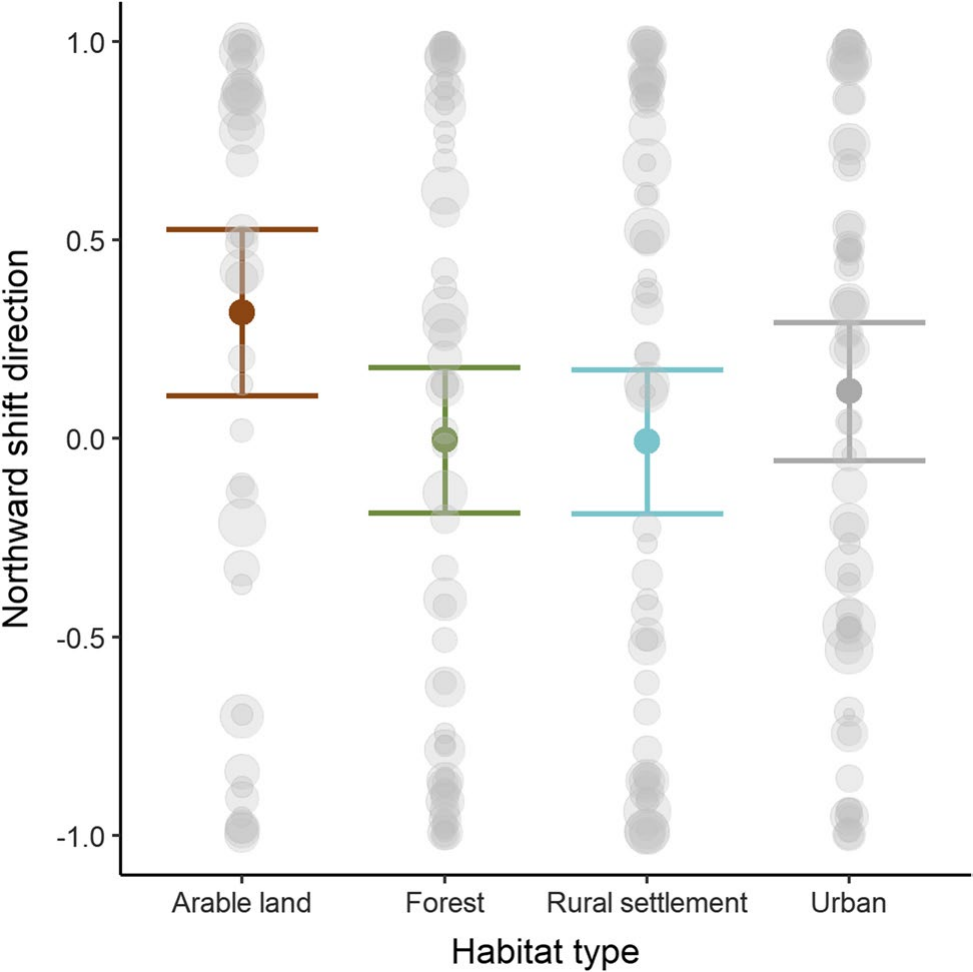
Predicted effects on northward shift direction in center of gravity per four habitat types based on the additive model (m2). Colored points show predicted means per habitat type with 95% confidence intervals (bars). Gray points represent the raw data, with varying size dependent on their weight given by the shift distance (see “Methods”). Higher northward values indicate northward shifts (+ 1) and lower values southward shifts (− 1). *N* = 234. Effect plot of snow period 2 is shown in ESM Fig. S10

**Fig. 3:**
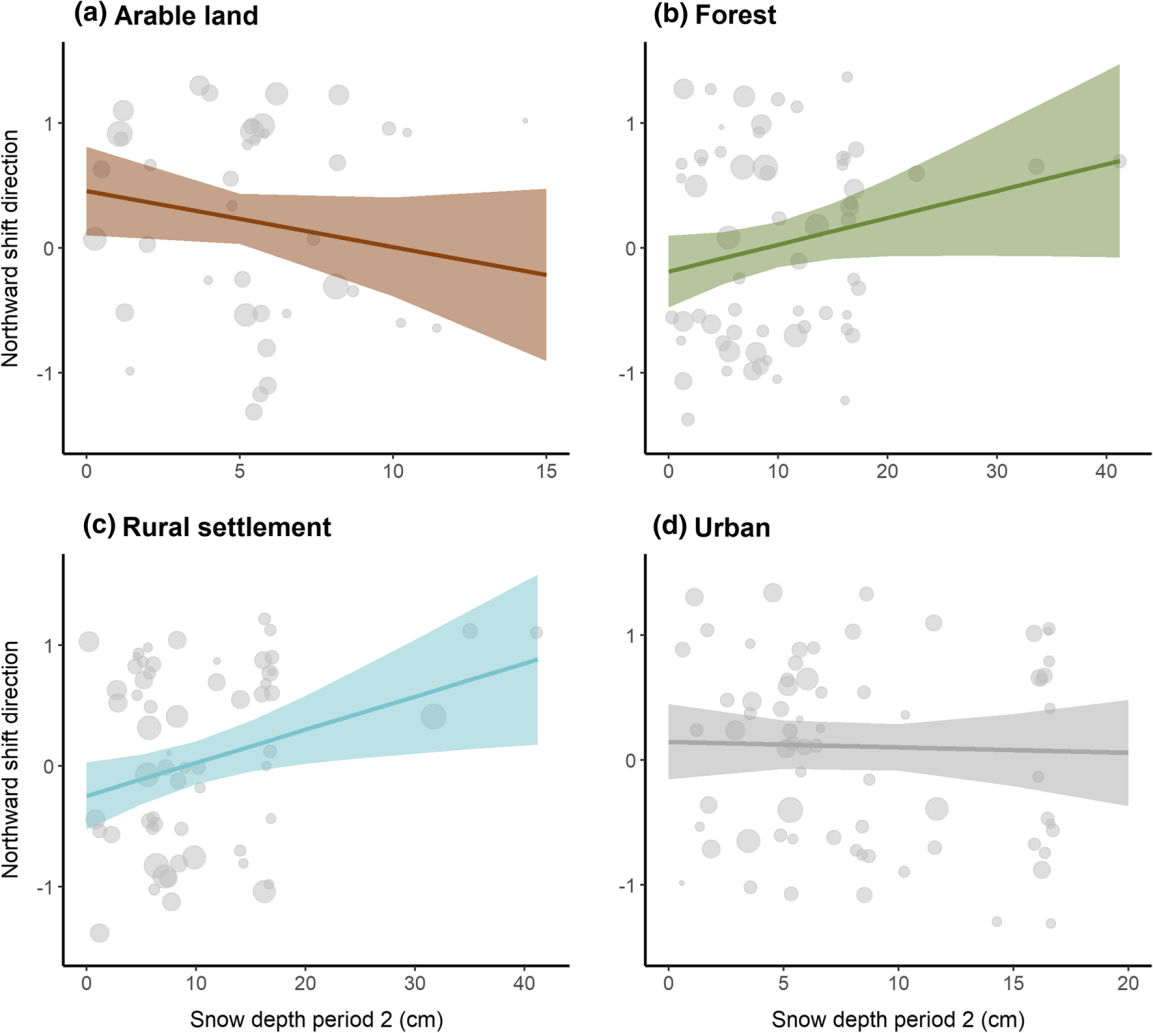
Predicted effects on direction of northward shifts based on the interaction model snow:habitat (m3), showing separate panels for **a** arable land, **b** forest, **c** rural settlement and **d** urban habitats. Regression lines show model estimates, shaded areas 95% confidence intervals. Gray points represent the raw data (i.e., one data point equals one species), with varying size dependent on their weight given by the shift distance (see “Methods”). Note the varying *x*-axis limits dependent on the raw data value ranges per habitat type. *N* = 234

Northward shift: The two top ranked models explaining northward shifts in center of gravity had very similar AIC values (Table 1) and are thus both presented (m2 and m3). Model 2 (habitat + snow p2) demonstrated that populations overwintering in arable land have significantly shifted northward, while forest and rural settlement species show no clear shift pattern along the latitudinal axis (Fig. 2, Table 1). The significant positive influence of snow depth in period 2 (Table 1) indicates that species which can tolerate snow rich areas have shifted more clearly northward overall compared to species which avoid snow (ESM Fig. S12). Yet in model 3 (habitat × snow p2) we demonstrate that the influence of snow on the northward shift direction depends on the habitat type based on a significant interaction of habitat with snow depth. This interaction shows that rural settlements and forest species had a significantly different slope from arable land species, with the latter being negatively related to snow depth while forest and rural settlement habitats showed a slight positive trajectory with snow depth (Fig. 3a–d). This means that in arable land, species preferring lower snow cover depths have moved northward compared to forests and rural settlement areas, where on the other hand shifts are stronger in species which can tolerate snow rich conditions.

### Shift differences among main vs. subhabitats and habitat specialization

Main Habitat: We found no difference in shift distance of northern range margins or northward shift in center of gravity between the main and subhabitats (ESM Table S3). However, there was a tendency that species’ center of gravity shifted less clearly eastward in their main habitats compared to the subhabitats (lmer: *b* = − 0.160 ± 0.087 SE, *t* value = − 1.849, *p* value = 0.066, conditional *R*^2^ = 0.208, *N* = 219, random intercept variance (species = 66 levels) = 0.129 ± 0.359, ESM Fig. S13).

**Fig. 4:**
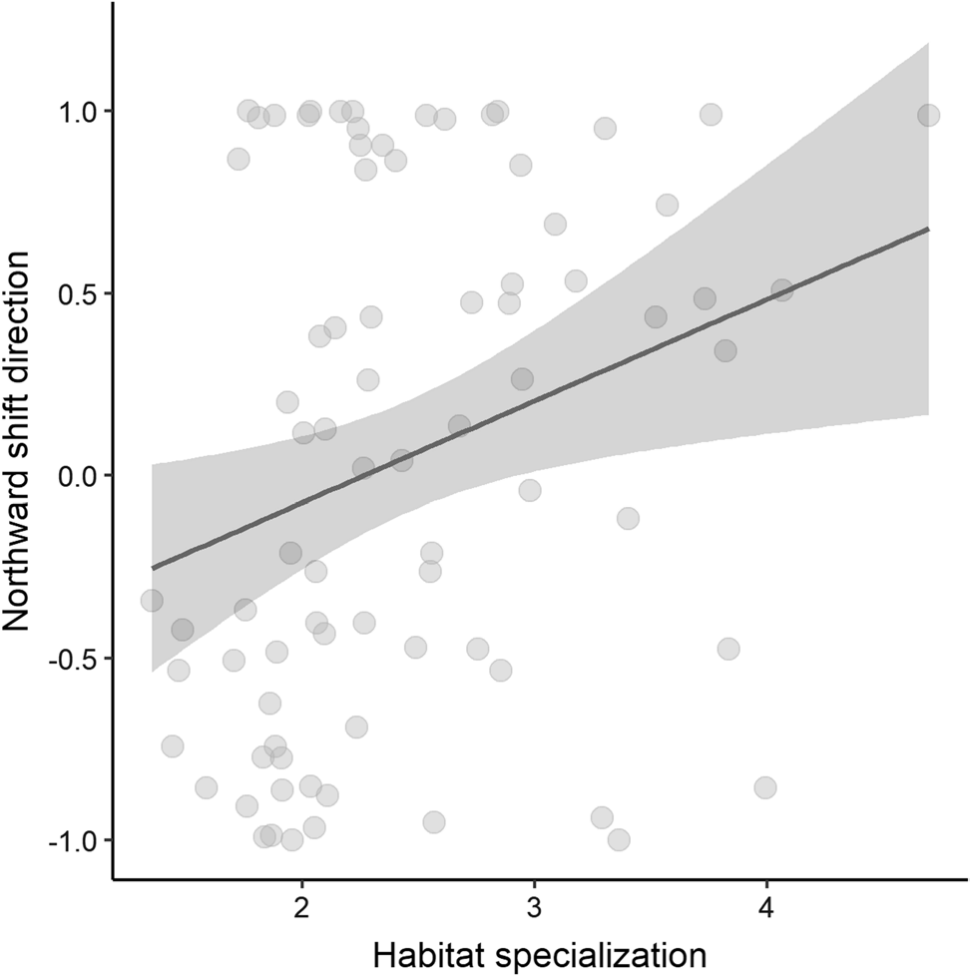
Predicted effects of habitat specialization (measured as relative densities in species’ main habitat types with higher values indicating stronger specialization) on northward shift direction. Regression lines show model estimates, shaded areas 95% confidence intervals and gray points the raw data. Higher northward values indicate northward shifts (+ 1), and lower values southward shifts (− 1). *N* = 80 (based on 80 species)

Habitat specialization: There was no apparent pattern for the habitat specialization regarding the northern margin shift distance and eastward shift direction (ESM Table S3). However, species with a higher habitat specialization shifted their center of gravity toward north more significantly than less specialized species (lm: *b* = 0.290 ± 0.114 SE, *t* value = 2.549, *p* value = 0.013, adjusted *R*^*2*^ = 0.077, *N* = 80, Fig. 4).

## Discussion

There is ample evidence demonstrating the significant impacts of climate change on species’ distributions across the globe, including different taxa, ecosystems, and climatic regions (e.g., Hickling et al. 2006; Mason et al. 2015). Yet, how these distribution shifts may differ among different habitat types—not only among but also within species—has rarely been studied before (e.g., Pavón-Jordán et al. 2019; Greig et al. 2017). In summary, our findings based on long-term observations of overwintering bird abundance and distribution emphasize the role of habitat use in climate-driven range shifts, as habitat type, species’ snow depth tolerance, and species’ habitat specialization all affect the pattern of species range shifts in winter. Our results also underline the general observation of movements toward northern directions using both northern range margin and abundance (measured by center of gravity) data. The only general shifts southward occurred in rural settlements and urban areas. This could be linked to the fact that the Finnish human population has been aggregated from rural areas to cities of Southern Finland (Official Statistics of Finland 2022).

Regarding shifts of the northern range margin, we found that the interaction model with habitat and snow depth tolerance performed best—similar to the northward shift directions. However, the response pattern for forest birds was opposite, with species preferring lower snow depths shifting their ranges northward farther (negative response), while it was positive for the northward shift direction (see below). Forests constitute the most natural habitat among the four studied habitat types here and likely differ the most from southern to northern parts of the country in terms of (winter) food availability. Hence, the food resources in forests are probably more limited in the North where fewer people live (i.e., less bird feeders) and natural resources such as insects and berries are not available at those latitudes during harsh winters of high snowfall. In addition, among the four habitat types, forests occur furthest north, which reinforces the above argumentation of limited resources during harsh winters as compared to habitats whose range limits are further south.

When exploring northward shifts in the center of gravity, we find a significant impact of both the additive model (single terms of snow tolerance and habitat) and interaction model with an interaction term between snow depth tolerance and habitat. First, with the interaction effect we see that shifts in rural settlements and forest habitats are more directional toward north in species with high snow depth tolerance compared to arable lands and urban habitats (Fig. 3). This could indicate that species with higher snow depth tolerance benefit from climate change in rural settlements and forest areas, which typically still have snow cover in most parts of the country. Species that are tolerant to deep snow conditions are adapted to forage in forests and feeders of the settlements, but still milder winters may increase their survival (Askeyev et al. 2018). Additionally, high snow depth conditions quite naturally have stronger impacts in open areas (i.e., arable lands) as compared to closed habitats (forests) where birds may feed in winter, e.g., on seeds and buds of trees, or dormant insects in tree barks. On the other hand, poleward shifts were more clearly northward in arable lands overall, and in species preferring low snow depth, as compared to the other habitats. This could be because overall the access to food has improved in open arable lands due to lower snow depths resulting from climate change. The importance of arable lands to overwintering birds has been previously highlighted (e.g., Goławski and Kasprzykowski 2010; Henderson et al. 2004). Here, we show that arable lands not only provide overwintering foraging sites to birds but also potentially aid their shifts poleward. Contrastingly to eastward shifts, we see a positive effect of snow depth in northward shifts in the additive model. Quite likely, the overall shift pattern with snow depth across all four habitats is dominated by rural settlements and forests, which showed a positive relationship with snow depths (see above and Fig. 3), and together dominated the dataset as compared to arable lands (see “Methods”), hence overruling the negative relationship observed in arable lands (no clear pattern detected in urban habitats).

When it comes to eastward shifts in the center of gravity, we find that shifts are more directional toward east in species preferring low snow depth (negative relationship). In Finland, there is a strong decreasing gradient in snow depth in southern and central Finland from west to east (Luomaranta et al. 2019, ESM Fig. S15). Hence, decreasing snow depth in these areas could favor eastward shifts especially in species which cannot tolerate higher snow depths.

We observed a more directional northward shift in species with a higher habitat specialization, indicating that species with less flexibility in using different habitats may respond to climatic warming more strongly by shifting their wintering ranges toward north. This result underlines similar earlier findings stating that more specialized species disperse farther and are more migratory (Martin and Fahrig 2018), given that life history traits such as dispersal and migration behavior are linked to species’ range shifts (e.g., Williams and Blois 2018). On the other hand, we did not find clear support that species would move differently in their main preferred habitat compared to subhabitats, although it has been found that species using different habitats may shift differently throughout their annual cycle, as habitat type also explains the variation in the directional pattern of range shifts in breeding birds (Lehikoinen and Virkkala 2016).

There is a growing body of evidence showing that species traits explain the variation in their response to climate change (MacLean and Beissinger 2017; Pacifici et al. 2017), to which this study contributes, and we thus suggest taking habitat-relevant but also weather-related traits, such as snow depth tolerance, into account. Species traits that relate to habitat use could indirectly shape the pattern of range shifts through interactions between climate and land cover changes, where, e.g., neotropical and temperate migratory birds show contrasting range shift patterns (Rushing et al. 2020). Such traits also affect the abundance trends of birds. For example, species with smaller breadth of diets are known to shift poleward faster (Auer and King 2014), and migratory distance and the size of the non-breeding grounds explain population trends of migratory birds, as they are relevant to the birds’ exposure to habitat loss and climate change (Patchett et al. 2018).

Although we assume the detectability of birds to be similarly high across habitats and years, it is possible that detection probability is higher in more open or semi-open habitats as compared to closed habitats such as forests, whereas many bird observations are done auditorily and thus depend less on visibility. In our study, we compared species-specific shifted distances and directions among habitats derived from relative abundances, but did not compare abundances among habitats directly and thus a systematic bias in our results is unlikely. Nevertheless, it is important to keep this detection probability caveat in mind especially when planning and conducting more research investigating habitat-specific species responses. Together, our results underline that habitat and climate interactively drive species range shifts, and thus season-specific habitat factors should be considered when investigating climate-driven range shifts, especially when studying overwintering populations. Also, species tend to show lower site fidelity in winter and could thus shift their distributions farther compared to the breeding season, as shown, e.g., for European and North American winter bird communities that have been changing faster toward a dominance of warm-dwelling species than breeding communities (Lehikoinen et al. 2021a, b).

## Supplementary Information

Below is the link to the electronic supplementary material.

Supplementary file1 (DOCX 3873 KB)

## Data Availability

The raw data used for this study is publicly available and can be downloaded from Laji.fi: https://laji.fi/observation/list?collectionId=HR.39

## Code availability

The code and part of the data to calculate the center of gravity range shifts are available at zenodo https://doi.org/10.5281/zenodo.6592592
