# Supplementary material for: Range shifts of overwintering birds depend on habitat type, snow conditions and habitat specialization
Source: Oecologia. 2022 Jun 29;199(3):725–36. doi: 10.1007/s00442-022-05209-5 (PMC9309152; doi:10.1007/s00442-022-05209-5)
Supplement: Supplementary file 1 — Supplementary file1 (DOCX 3873 KB) [file 442_2022_5209_MOESM1_ESM.docx]

Electronic Supplemental Material


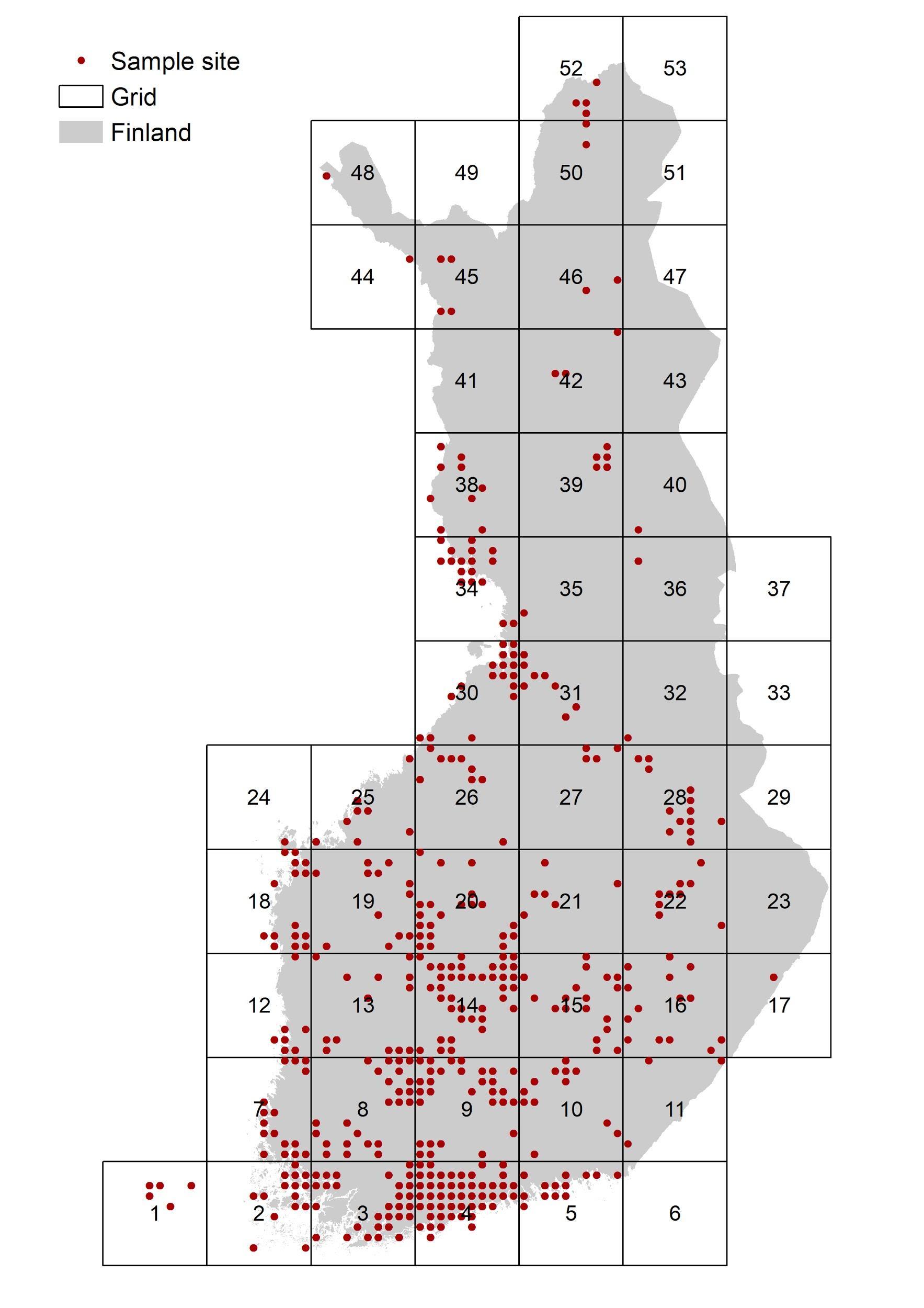


**Fig. S1.** Sampled transects and 100-km grids for analysis. The sample sites are geometric centres of the sampled transects.


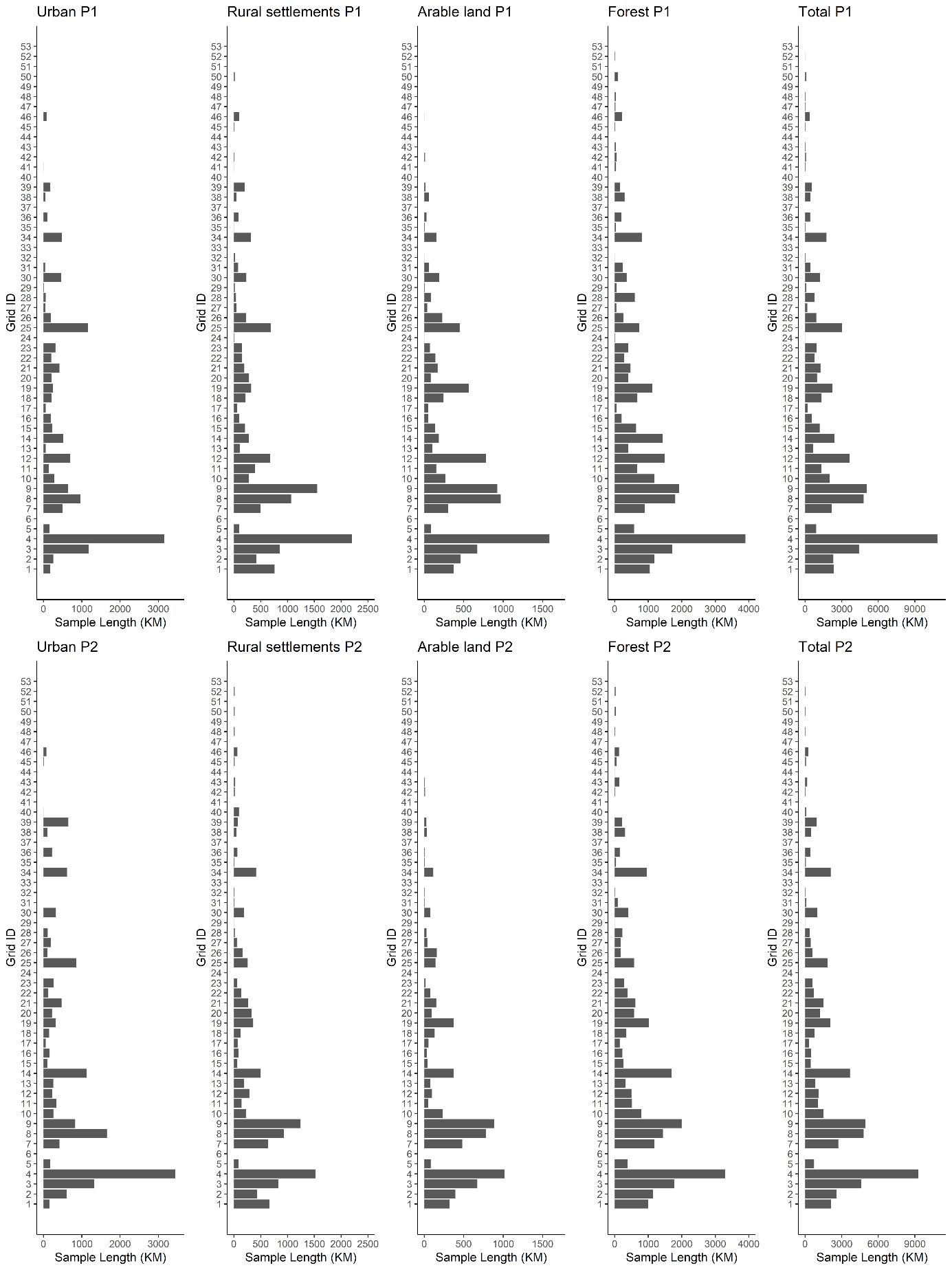


**Fig. S2.** A summary of the surveyed transect lengths in km per 100-km grid for the two periods (period 1: 1987-1999, period 2: 2010-2020).


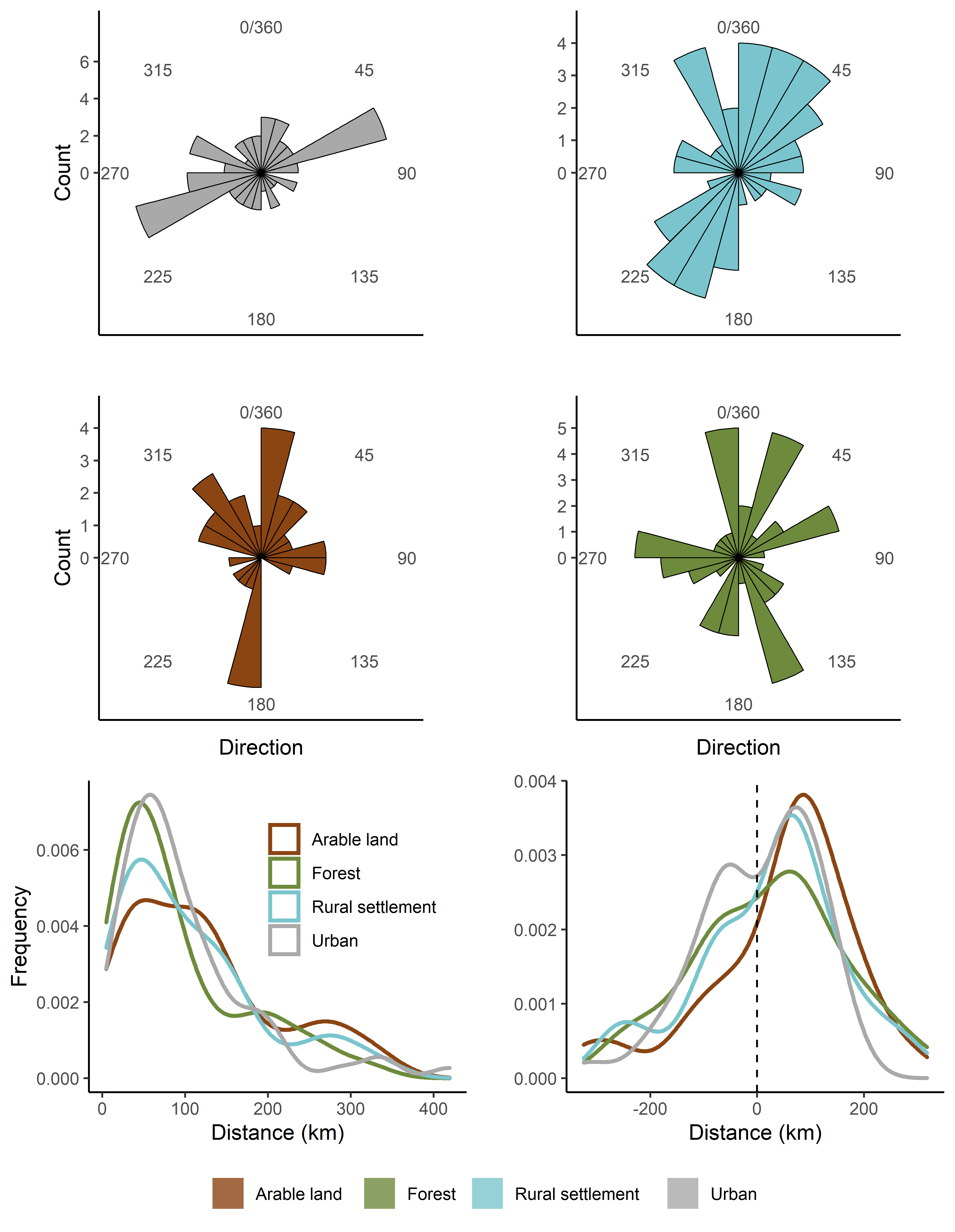


**Fig. S3.** Habitat-specific histograms of the range shift patterns for the shift direction (top four), and density plots for overall distance and latitudinal distance (bottom two).


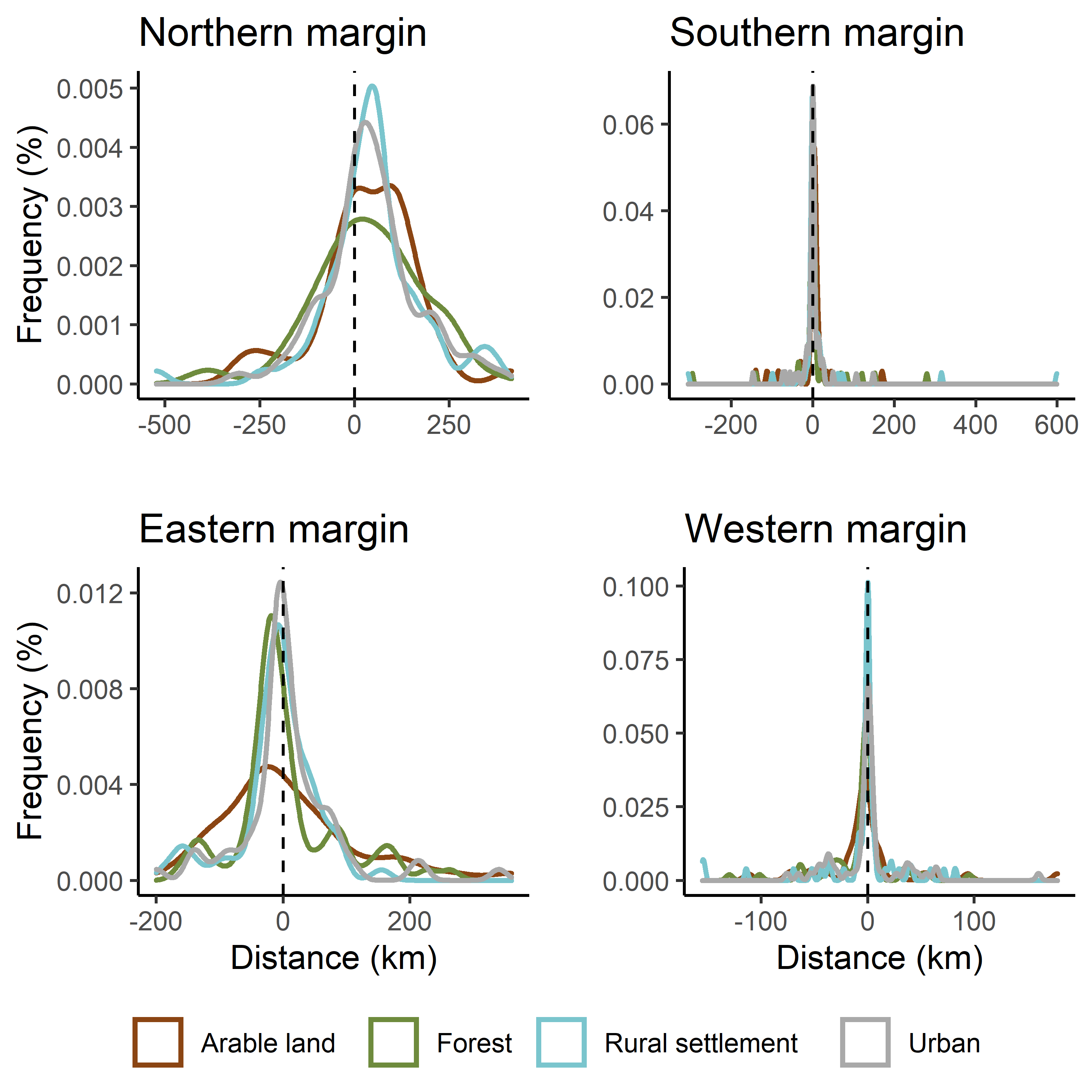


**Fig. S4.** Density plots of the margin shift distances for northern, southern, eastern and western margins. Northern margin shift distances was included as response variable in the modeling, while the other variables are presented for data description purposes.

**
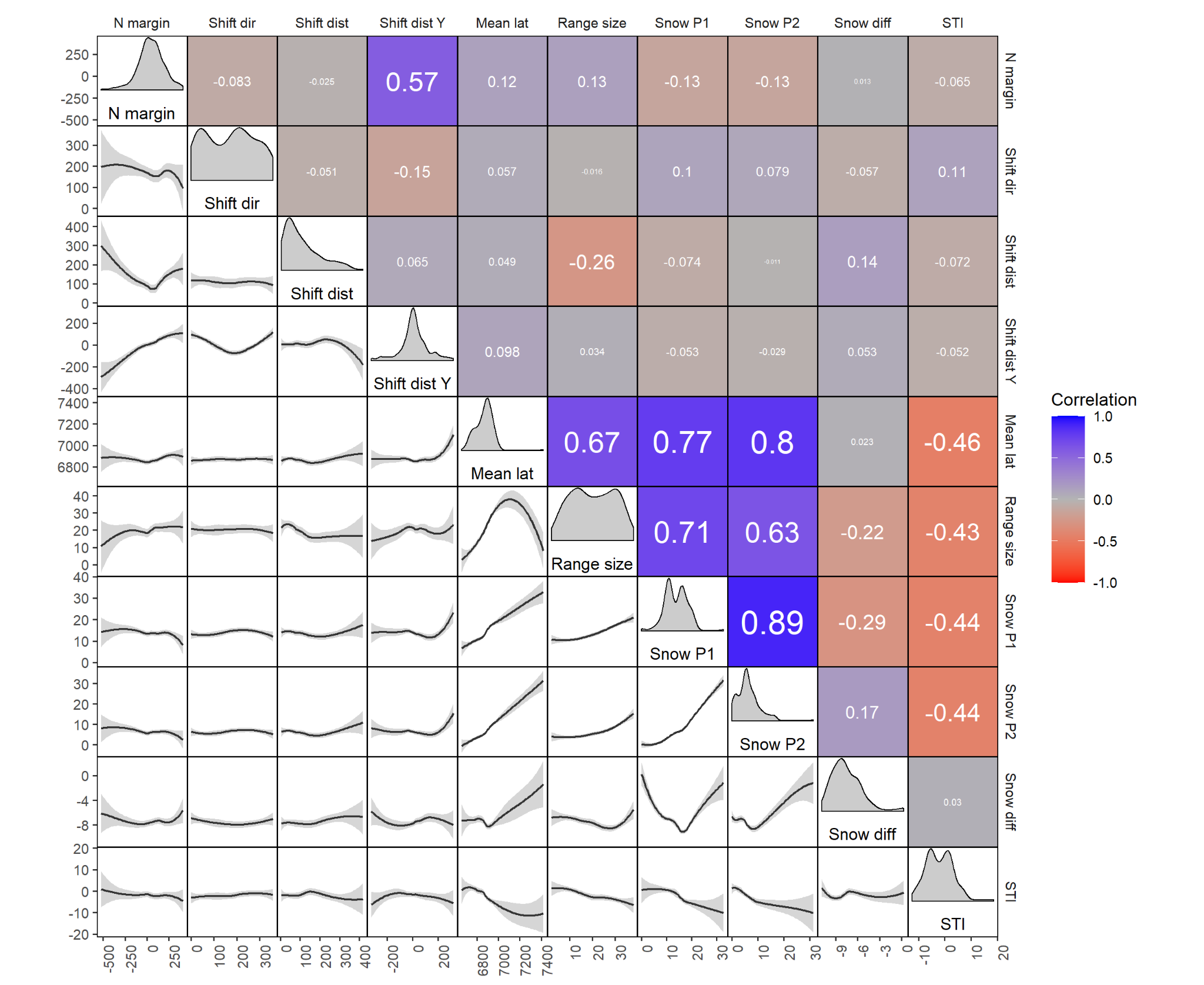
Fig. S5.** Spearman correlations of continuous predictors. Higher correlations are shown with larger figures, where blue colors represent positive and red colors negative correlations.

**Table S1.** Model outputs (AIC values, estimates, standard errors SE, t and p values) for the models performing worse than the top model for **northern range margin shift distances** (based on lowest AIC). We transformed the response variable to units of 100 km to avoid modeling large values. For each model, the sample size (N), AIC, AIC weight (w) and conditional *R^2^* are given. Significant and marginal effects (p<0.1) are depicted in bold. Continuous predictors were standardized prior to modeling. For the factor habitat, arable land was used as reference level in all models. Note that species-habitat combinations where the northern range was higher than 66.85°N latitude were excluded (N = 4 species, and 54 observations).

| **Term** | **Beta*** | **SE*** | **t-value** | **p-value** |
| --- | --- | --- | --- | --- |
| *m1: Northern margin ~ habitat, N = 180, AIC =649.56, AIC w = 0.125, R^2^ = 0.372* | | | | |
| Intercept | 0.339 | 0.225 | 1.508 | 0.133 |
| Forest | 0.161 | 0.276 | 0.584 | 0.560 |
| Rural settlement | 0.011 | 0.278 | 0.038 | 0.970 |
| Urban | 0.191 | 0.273 | 0.699 | 0.486 |
| Random: species (N = 77) | 0.834 | 0.913 |  |  |
| Random: residual | 1.421 | 1.192 |  |  |
| *m4: Northern margin ~ habitat + STI, N = 180, AIC = 650.61, AIC w = 0.074, R^2^ = 0.374* | | | | |
| Intercept | 0.317 | 0.225 | 1.408 | 0.161 |
| Forest | 0.171 | 0.275 | 0.622 | 0.535 |
| Rural settlement | 0.018 | 0.277 | 0.066 | 0.948 |
| Urban | 0.234 | 0.276 | 0.847 | 0.399 |
| STI | -0.132 | 0.135 | -0.976 | 0.332 |
| Random: species (N = 77) | 0.824 | 0.908 |  |  |
| Random: residual | 1.417 | 1.190 |  |  |
| *m5: Northern margin ~ habitat * STI, N = 180, AIC = 652.86, AIC w = 0.024, R^2^ = 0.372* | | | | |
| Intercept | 0.338 | 0.224 | 1.512 | 0.132 |
| Forest | 0.190 | 0.274 | 0.693 | 0.490 |
| Rural settlement | -0.001 | 0.276 | -0.005 | 0.996 |
| Urban | 0.254 | 0.276 | 0.921 | 0.358 |
| STI | 0.173 | 0.258 | 0.670 | 0.504 |
| Forest:STI | -0.146 | 0.312 | -0.469 | 0.640 |
| **Rural settlement:STI** | **-0.516** | **0.307** | **-1.683** | **0.095** |
| Urban:STI | -0.399 | 0.301 | -1.326 | 0.187 |
| Random: species (N = 77) | 0.775 | 0.880 |  |  |
| Random: residual | 1.404 | 1.185 |  |  |
| *m2: N margin ~ habitat + snow P2, N = 180, AIC = 648.12, AIC w = 0.257, R^2^ = 0.401* | | | | |
| Intercept | 0.275 | 0.226 | 1.220 | 0.224 |
| Forest | 0.232 | 0.274 | 0.846 | 0.399 |
| Rural settlement | 0.140 | 0.283 | 0.496 | 0.621 |
| Urban | 0.268 | 0.273 | 0.982 | 0.328 |
| **Snow period 2** | **-0.216** | **0.115** | **-1.872** | **0.063** |
| Random: species (N = 77) | 0.864 | 0.930 |  |  |
| Random: residual | 1.371 | 1.171 |  |  |

**For random effects (species), the variance and standard deviation are shown.*

**Table S2.** Model outputs (AIC values, estimates, standard errors SE, t and p values) for the models performing worse than the top model (based on lowest AIC) **for eastward** (m1, m2, m3, m5) and **northward** (m1, m2, m3) **shift directions**. For each model, the sample size (N), AIC, AIC weight (w) and conditional *R^2^* are given. Significant and marginal effects (p<0.1) are depicted in bold. Continuous predictors were standardized prior to modeling. For the factor habitat, arable land was used as reference level in all models.

| **Term** | **Beta*** | **SE*** | **t-value** | **p-value** |
| --- | --- | --- | --- | --- |
| *m1: eastward ~ habitat, N = 234, AIC = 539.338, AIC w = 0.081, R^2^ = 0.005* | | | | |
| Intercept | 0.019 | 0.099 | 0.190 | 0.850 |
| Forest | 0.089 | 0.116 | 0.767 | 0.444 |
| Rural settlement | 0.026 | 0.116 | 0.226 | 0.822 |
| Urban | -0.076 | 0.114 | -0.667 | 0.505 |
| Random: species (N = 81) | 0.158 | 0.398 |  |  |
| Random: residual | 29.377 | 5.420 |  |  |
| *m4: eastward ~ habitat + STI, N = 234, AIC = 539.947, AIC w = 0.059, R^2^ = 0.005* | | | | |
| Intercept | 0.019 | 0.099 | 0.189 | 0.850 |
| Forest | 0.080 | 0.116 | 0.685 | 0.494 |
| Rural settlement | 0.021 | 0.116 | 0.184 | 0.854 |
| Urban | -0.064 | 0.114 | -0.562 | 0.575 |
| STI | -0.065 | 0.055 | -1.182 | 0.240 |
| Random: species (N = 81) | 0.151 | 0.389 |  |  |
| Random: residual | 30.299 | 5.505 |  |  |
| *m5: eastward ~ habitat * STI, N = 234, AIC = 542.050, AIC w = 0.021, R^2^ = 0.006* | | | | |
| Intercept | 0.025 | 0.099 | 0.249 | 0.804 |
| Forest | 0.083 | 0.116 | 0.718 | 0.474 |
| Rural settlement | 0.004 | 0.115 | 0.038 | 0.970 |
| Urban | -0.082 | 0.113 | -0.725 | 0.469 |
| STI | -0.211 | 0.113 | -1.868 | **0.063** |
| Forest:STI | 0.206 | 0.125 | 1.650 | 0.100 |
| Rural settlement:STI | 0.074 | 0.133 | 0.554 | 0.580 |
| Urban:STI | 0.187 | 0.126 | 1.486 | 0.139 |
| Random: species (N = 81) | 0.158 | 0.398 |  |  |
| Random: residual | 29.377 | 5.420 |  |  |
| *m3: eastward ~ habitat * snow P2, N = 234, AIC = 538.311, AIC w = 0.135, R^2^ = 0.006* | | | | |
| Intercept | -0.141 | 0.163 | -0.869 | 0.386 |
| Forest | 0.263 | 0.172 | 1.533 | 0.127 |
| Rural settlement | 0.200 | 0.174 | 1.149 | 0.252 |
| Urban | 0.080 | 0.173 | 0.463 | 0.644 |
| Snow P2 | -0.262 | 0.211 | -1.242 | 0.216 |
| Forest:snow P2 | 0.056 | 0.218 | 0.259 | 0.796 |
| Rural settlement:snow P2 | 0.198 | 0.216 | 0.917 | 0.360 |
| Urban:snow P2 | 0.102 | 0.223 | 0.459 | 0.647 |
| Random: species (N = 81) | 0.160 | 0.400 |  |  |
| Random: residual | 28.730 | 5.460 |  |  |
| *m1: northward ~ habitat, N = 234, AIC = 553.040, AIC w = 0.115, R^2^ = 0.007* | | | | |
| Intercept | 0.259 | 0.103 | 2.509 | **0.013** |
| Forest | -0.246 | 0.118 | -2.084 | **0.038** |
| Rural settlement | -0.235 | 0.118 | -1.997 | **0.047** |
| Urban | -0.130 | 0.115 | -1.130 | 0.260 |
| Random: species (N = 81) | 0.201 | 0.448 |  |  |
| Random: residual | 30.664 | 5.538 |  |  |
| *m4: northward ~ habitat + STI, N = 234, AIC = 554.151, AIC w = 0.066, R^2^ = 0.007* | | | | |
| Intercept | 0.259 | 0.103 | 2.514 | **0.013** |
| Forest | -0.253 | 0.118 | -2.143 | **0.033** |
| Rural settlement | -0.238 | 0.117 | -2.026 | **0.044** |
| Urban | -0.121 | 0.115 | -1.046 | 0.297 |
| STI | -0.056 | 0.059 | -0.945 | 0.347 |
| Random: species (N = 81) | 0.198 | 0.445 |  |  |
| Random: residual | 30.662 | 5.534 |  |  |
| *m5: northward ~ habitat * STI, N = 234, AIC = 558.955, AIC w = 0.006, R^2^ = 0.007* | | | | |
| Intercept | 0.248 | 0.103 | 2.399 | **0.017** |
| Forest | -0.256 | 0.119 | -2.155 | **0.032** |
| Rural settlement | -0.233 | 0.118 | -1.978 | **0.049** |
| Urban | -0.119 | 0.116 | -1.028 | 0.305 |
| STI | 0.011 | 0.118 | 0.092 | 0.927 |
| Forest:STI | -0.123 | 0.128 | -0.963 | 0.337 |
| Rural settlement:STI | -0.096 | 0.137 | -0.703 | 0.483 |
| Urban:STI | -0.039 | 0.130 | -0.302 | 0.763 |
| Random: species (N = 81) | 0.198 | 0.445 |  |  |
| Random: residual | 30.417 | 5.515 |  |  |

**For random effect (species), the variance and standard deviation are shown.*

**Model diagnostics for eastward and northward shift direction:**

We visually checked model performance using the R function check_model (package see, Makowski et al. 2020, Fig. S6 and S7) and tested for normality of residuals, since we fitted linear mixed effect models which assume normal distribution of the residuals (R function check_normality, package performance, Lüdecke et al. 2021). We did this for the best model (m4 for both eastward and northward shift), finding a p value of 0.835 for eastward and 0.147 for northward shifts.


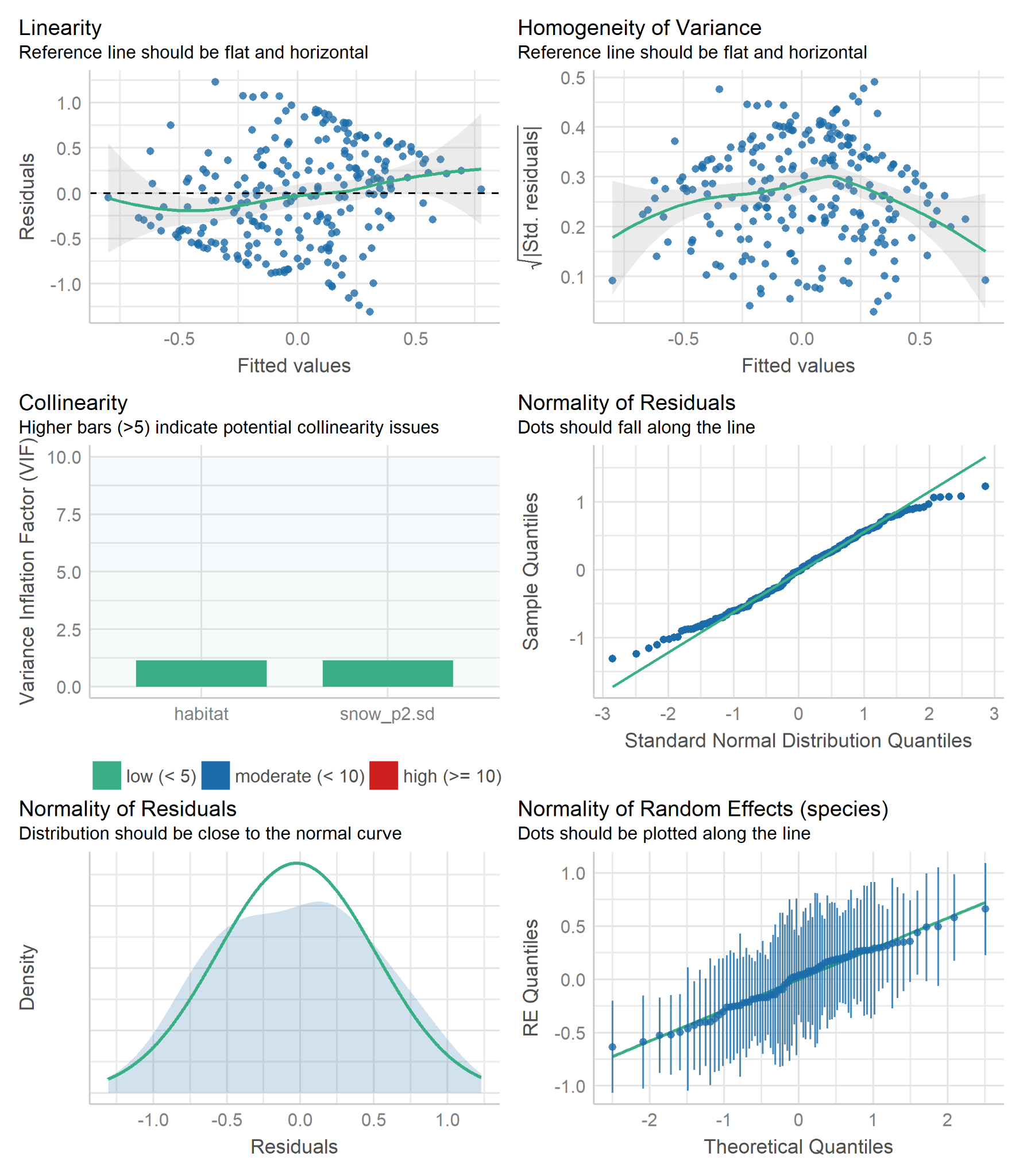


**Figure S6.** Model diagnostics for **eastward shift directions** against habitat and snow (m2).


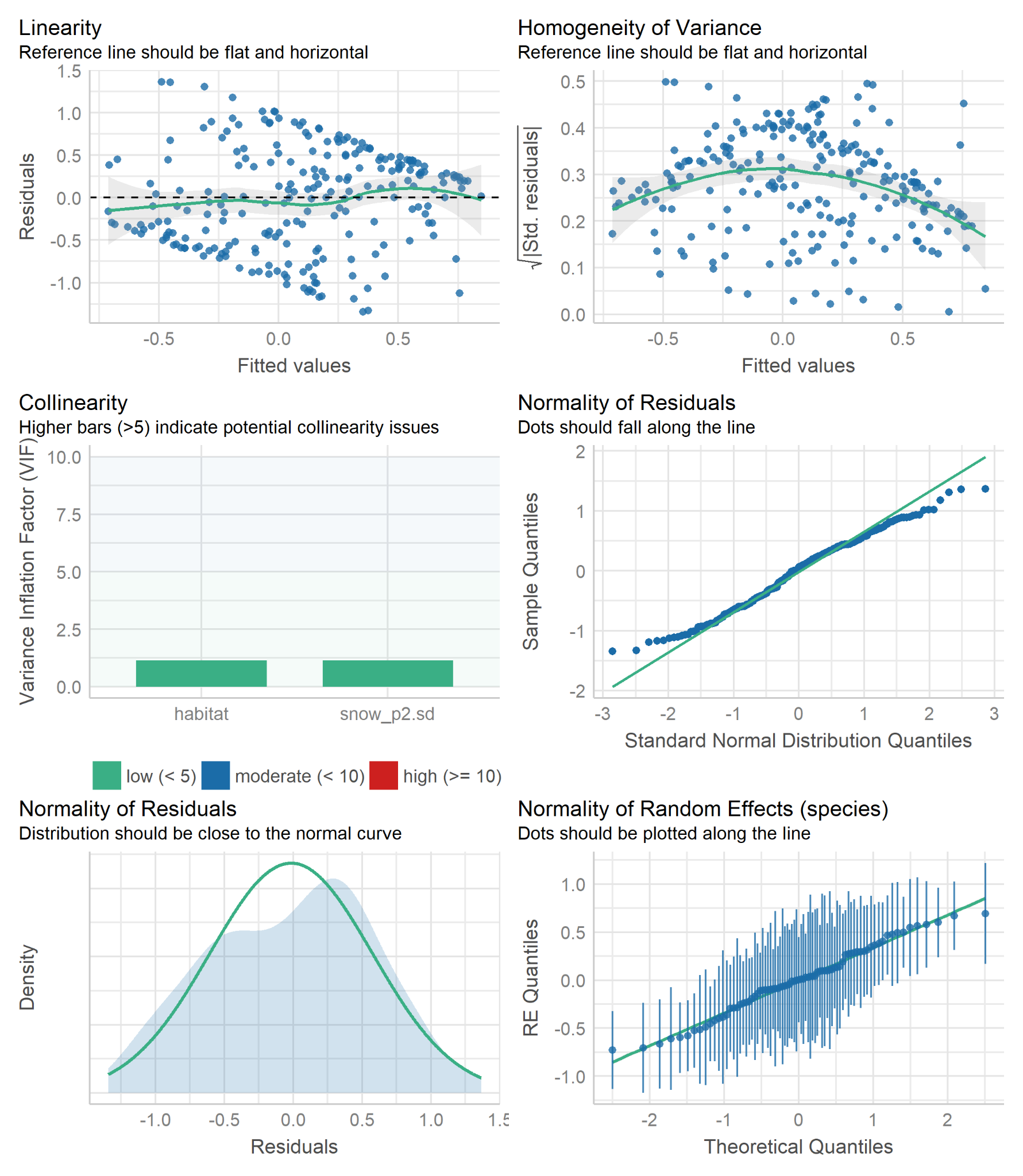


**Figure S7.** Model diagnostics for **northward shift directions** against habitat and snow (m2).


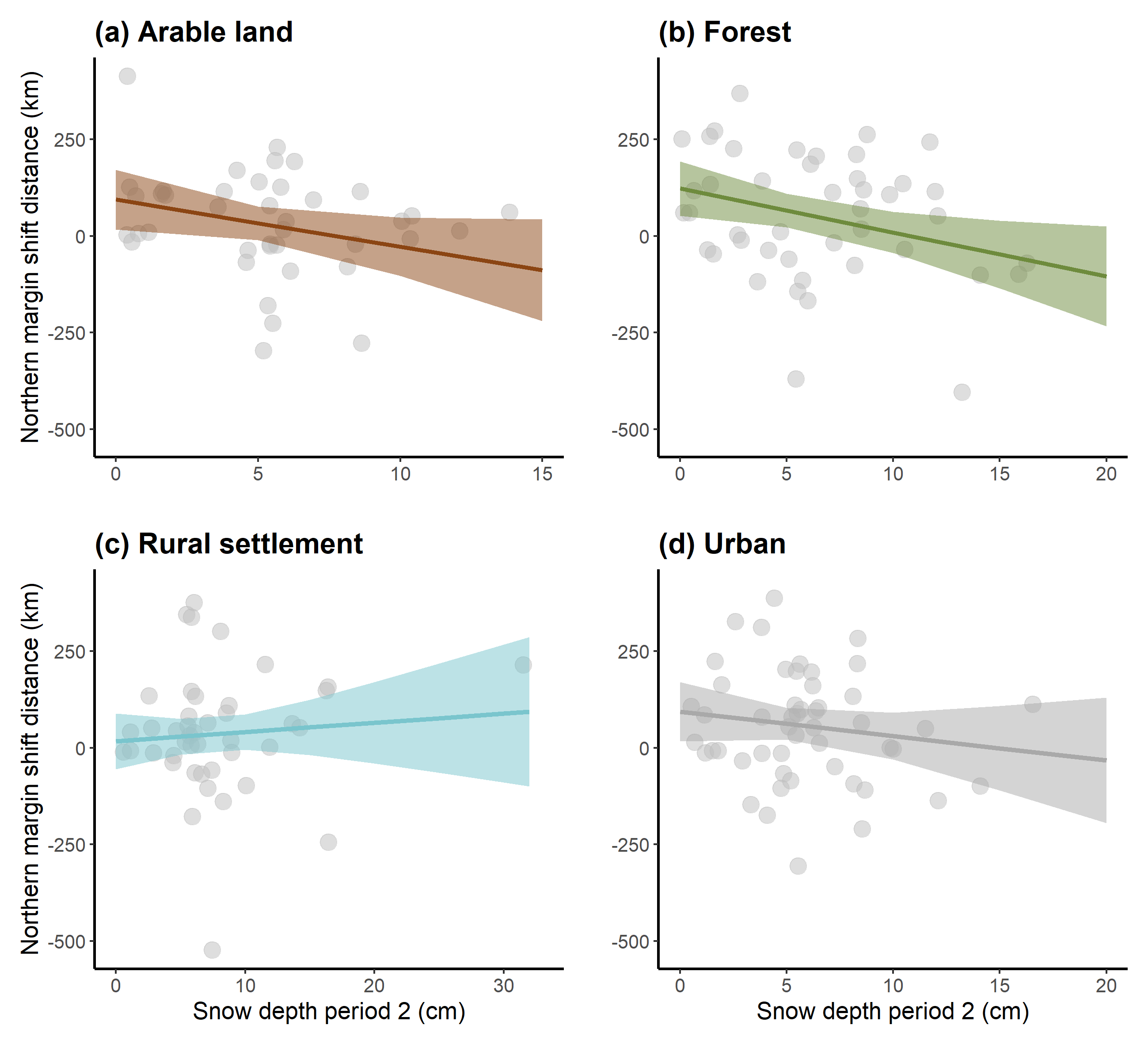


**Fig. S8.** Predicted effects on shift distances of northern range margins in relation to snow depth in period 2 (cm) in (a) arable land, (b) forest, (c) rural settlements and (d) urban habitats. Regression lines show model estimates, shaded areas 95% confidence intervals and points the raw data. Note the varying x-axes limits adjusted to the raw data coverage per habitat type. Species-habitat combinations where the northern range was higher than 66.85°N latitude were excluded (N = 4 species including 54 grid observations). N = 180.


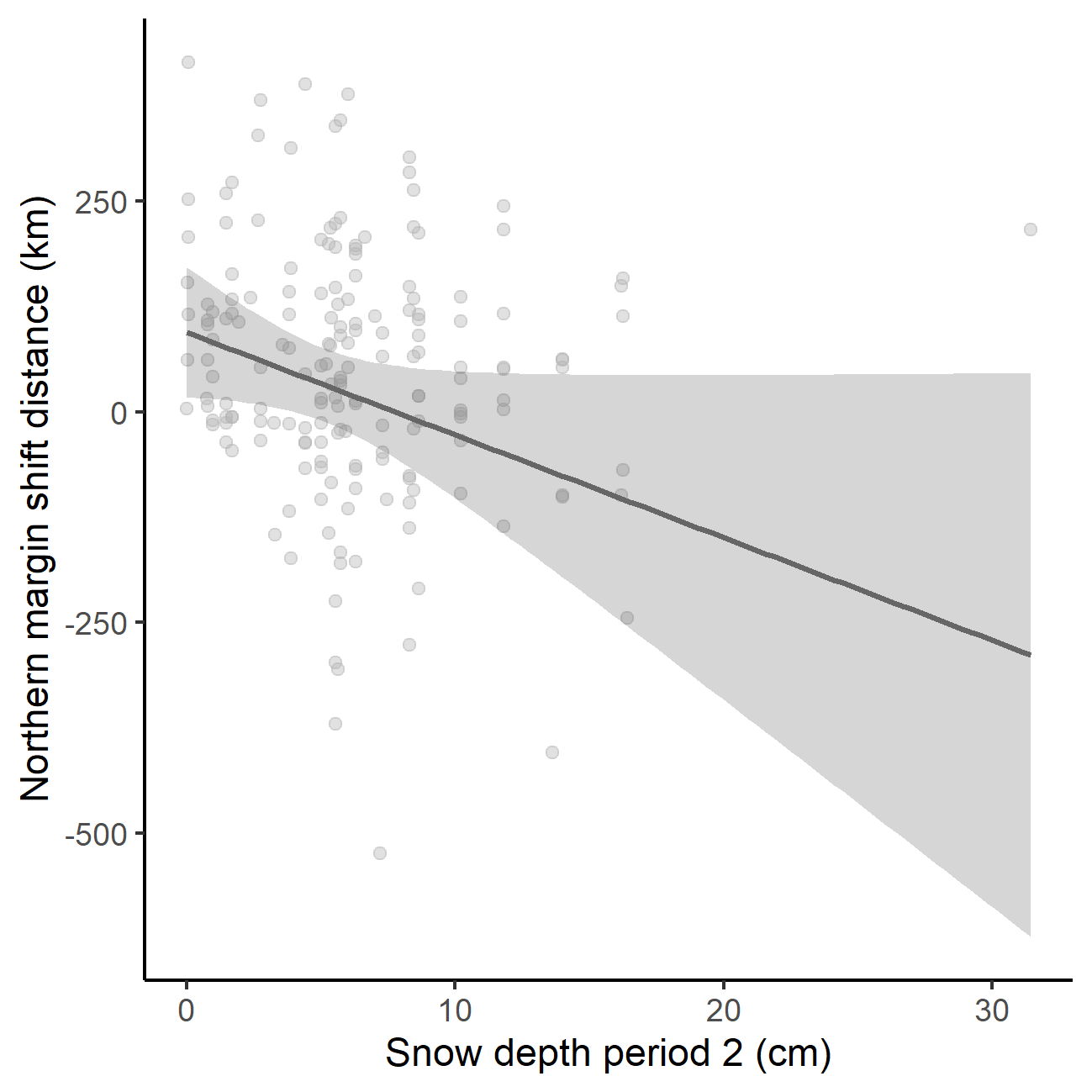


**Fig. S9.** Effect plot for the single term of snow depth in period 2 on the shift distance of northern range margins (model m5). Regression line depicts the mean predicted effects, grey area the 95% confidence interval and grey points the raw data (no model weights were specified here, thus the points have the same size). Positive shift distances indicate northward, negative values indicate southward shifts.


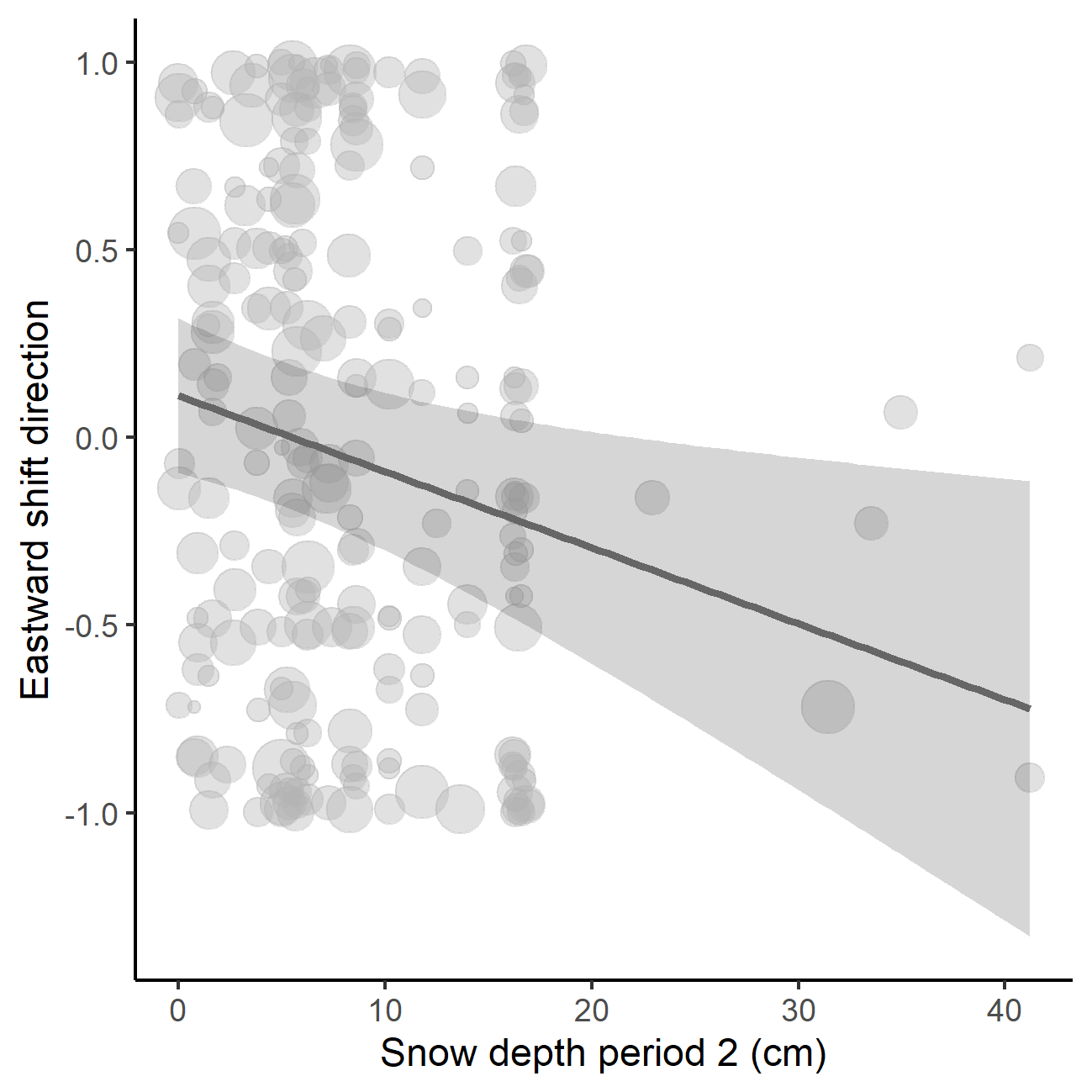


**Fig. S10.** Effect plot for the single term of snow depth in period 2 on the eastward shift direction (significant effect, model m4). Regression line depicts the mean predicted effects, grey area the 95% confidence interval. Grey points represent the raw data, with varying size dependent on their weight given by the shift distance (see methods).


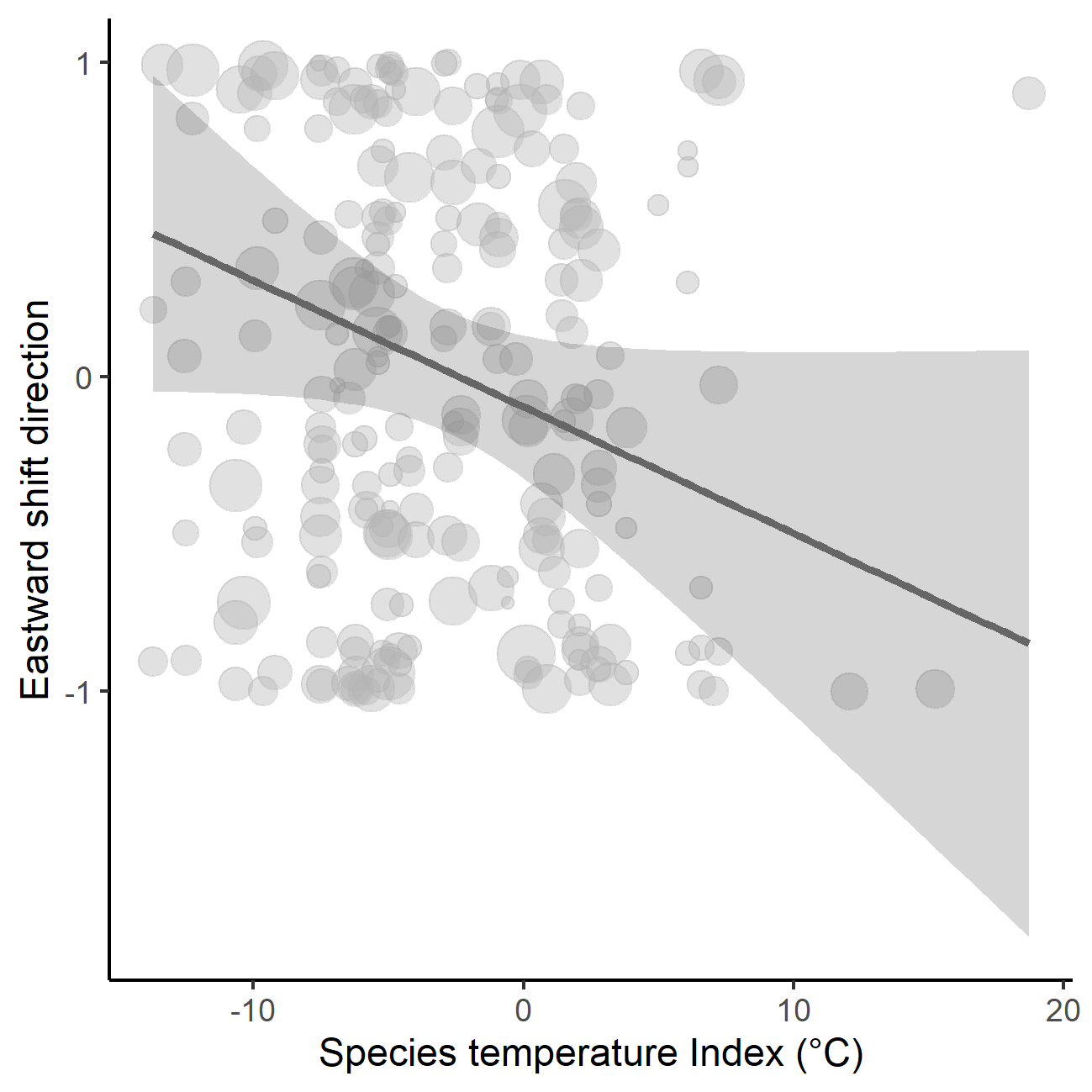


**Fig. S11.** Effect plot for the single term of STI on the eastward shift direction (marginal effect, model m3). Regression line depicts the mean predicted effects, grey area the 95% confidence interval. Grey points represent the raw data, with varying size dependent on their weight given by the shift distance (see methods).


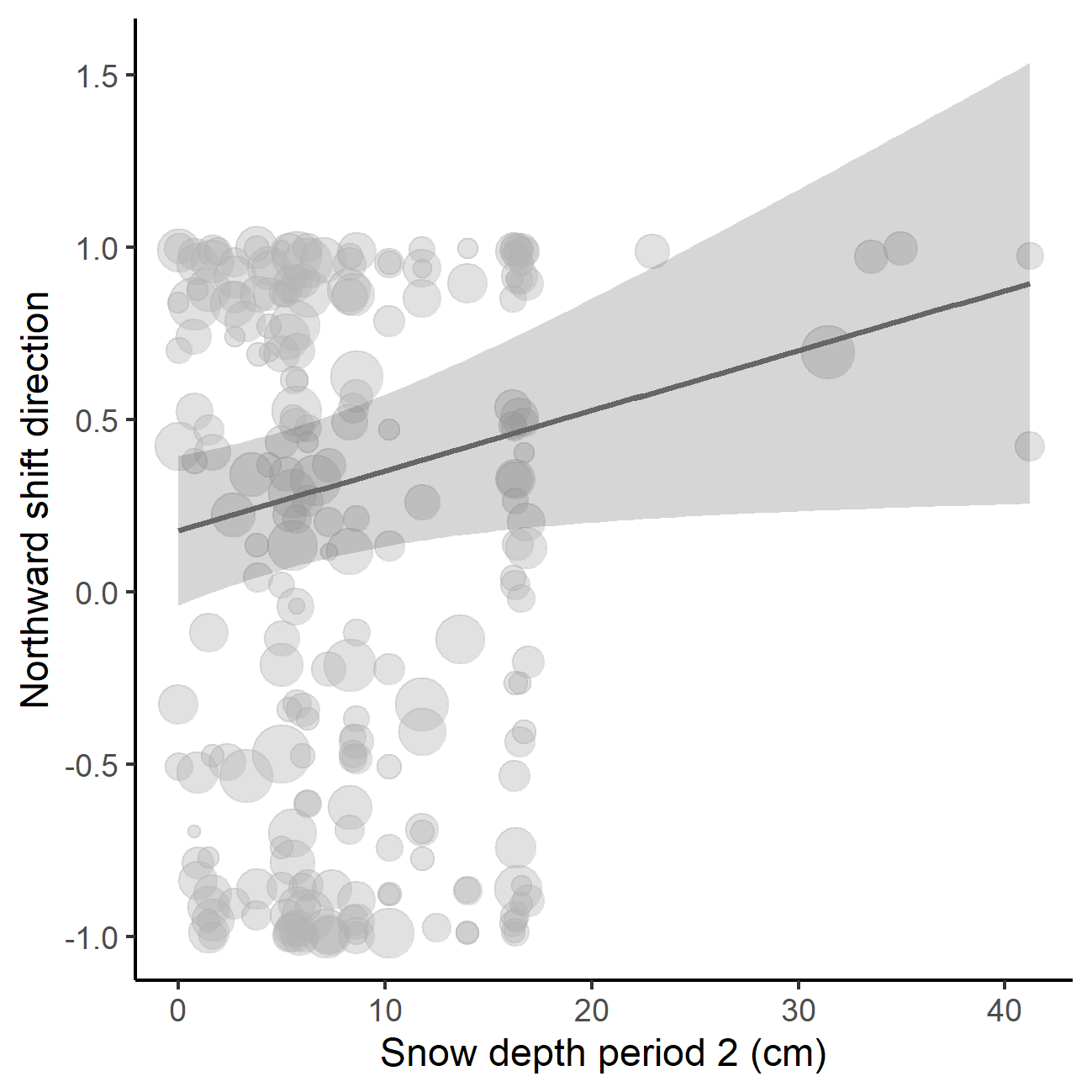


**Fig. S12.** Effect plot for the significant single term of snow depth in period 2 on the northward shift direction (model m4). Regression line depicts the mean predicted effects, grey area the 95% confidence interval. Grey points represent the raw data, with varying size dependent on their weight given by the shift distance (see methods).

**Table S3.** Model outputs (sample sizes, 𝛽-estimates, standard errors SE, t and p values) for the models testing differences **between main *vs*. sub habitats** (first two models, lmer) and **species’ habitat specialization** (measured as relative density in their main habitats, lm). *Note that species-habitat combinations where the northern range was higher than 66.85°N latitude were excluded (N = 2 species, and 54 observations) for northern range margin shift models and thus AIC values are shown as they are not comparable with northward and eastward shift direction models. Also, range margin shift was transformed to units of 100 km for better modeling of smaller values.

| **Term** | **Estimate**^**^ | **SE**^**^ | **t-value** | **p-value** |
| --- | --- | --- | --- | --- |
| **Shift northern margin ~ main habitat, N = 167, R^2^ = 0.361* | | | | |
| **Intercept** | **0.451** | **0.159** | **2.840** | **0.006** |
| Habitat: main | 0.027 | 0.219 | 0.125 | 0.901 |
| Random: species (N = 64) | 0.809 ^✢^ | 0.899 ^✢^ |  |  |
| Random: residual | 1.433 ^✢^ | 1.197 ^✢^ |  |  |
| *Northward shift ~ main habitat, N = 219, R^2^ = 0.232* | | | | |
| Intercept | 0.100 | 0.070 | 1.429 | 0.156 |
| Habitat: main | -0.049 | 0.097 | -0.501 | 0.617 |
| Random: species (N = 66) | 0.129 ^✢^ | 0.359 ^✢^ |  |  |
| Random: residual | 0.429 ^✢^ | 0.655 ^✢^ |  |  |
| **Shift northern margin ~ habitat specialization, N = 80, R^2^ = 0.013* | | | | |
| Intercept | 0.880 | 0.380 | 0.232 | 0.818 |
| Habitat specialization | 0.224 | 0.267 | 0.838 | 0.406 |
| *Eastward shift ~ habitat specialization, N = 80, R^2^ = 0.013* | | | | |
| Intercept | -0.208 | 0.154 | -1.349 | 0.181 |
| Habitat specialization | 0.121 | 0.118 | 1.025 | 0.309 |

*^**^For random effects (species), the variance and standard deviation are shown.*

**
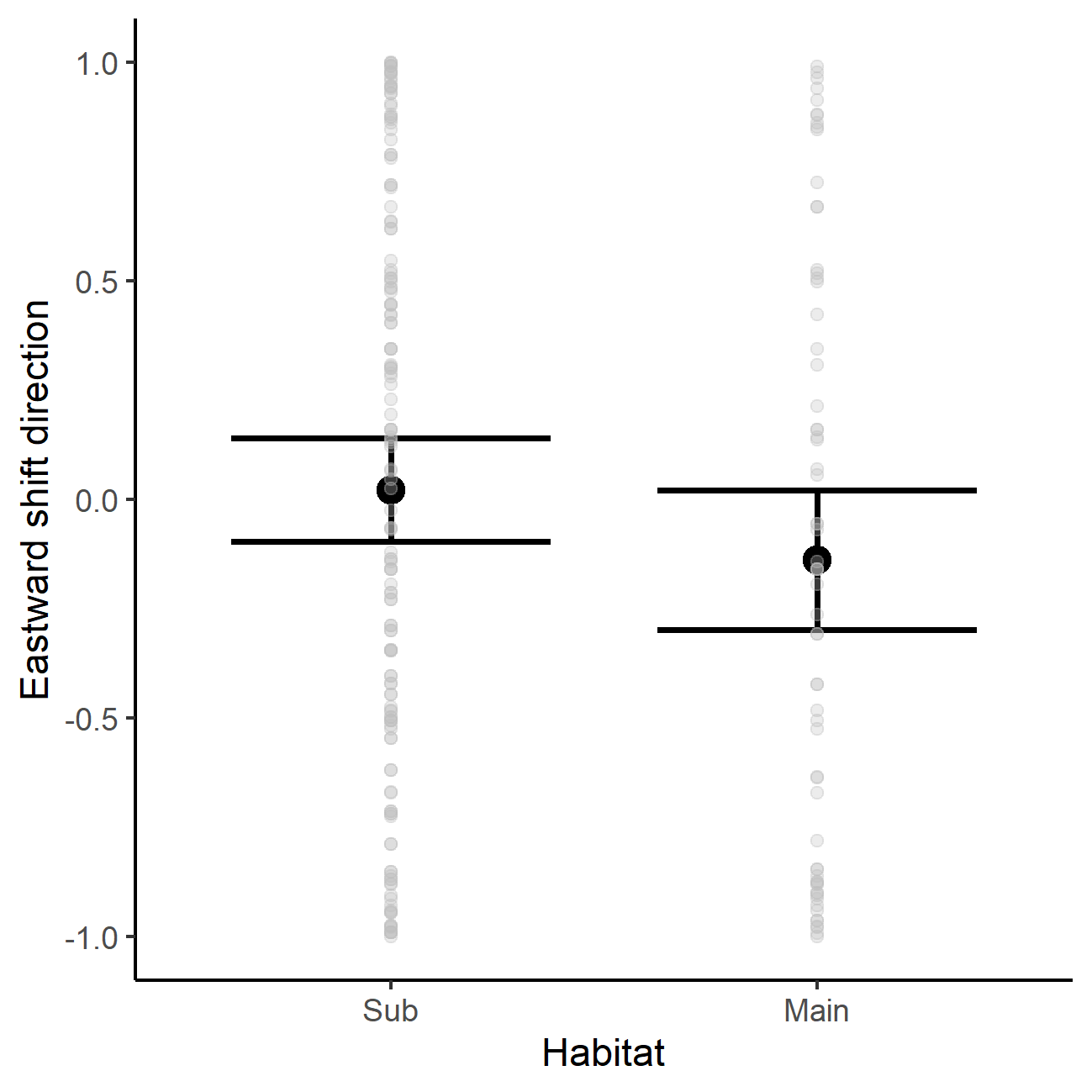
**

**Fig. S13.** Effect plot for the marginal difference between main and sub habitats with respect to species’ eastward shift directions in their center of gravity. Black dots depict the mean predicted effects, error bars the 95% confidence interval and grey points the raw data. Shift values at -1 represent westward direction and values +1 represent eastward direction. N = 219.

**Sensitivity Analyses:**

1. **Survey effort per grid**


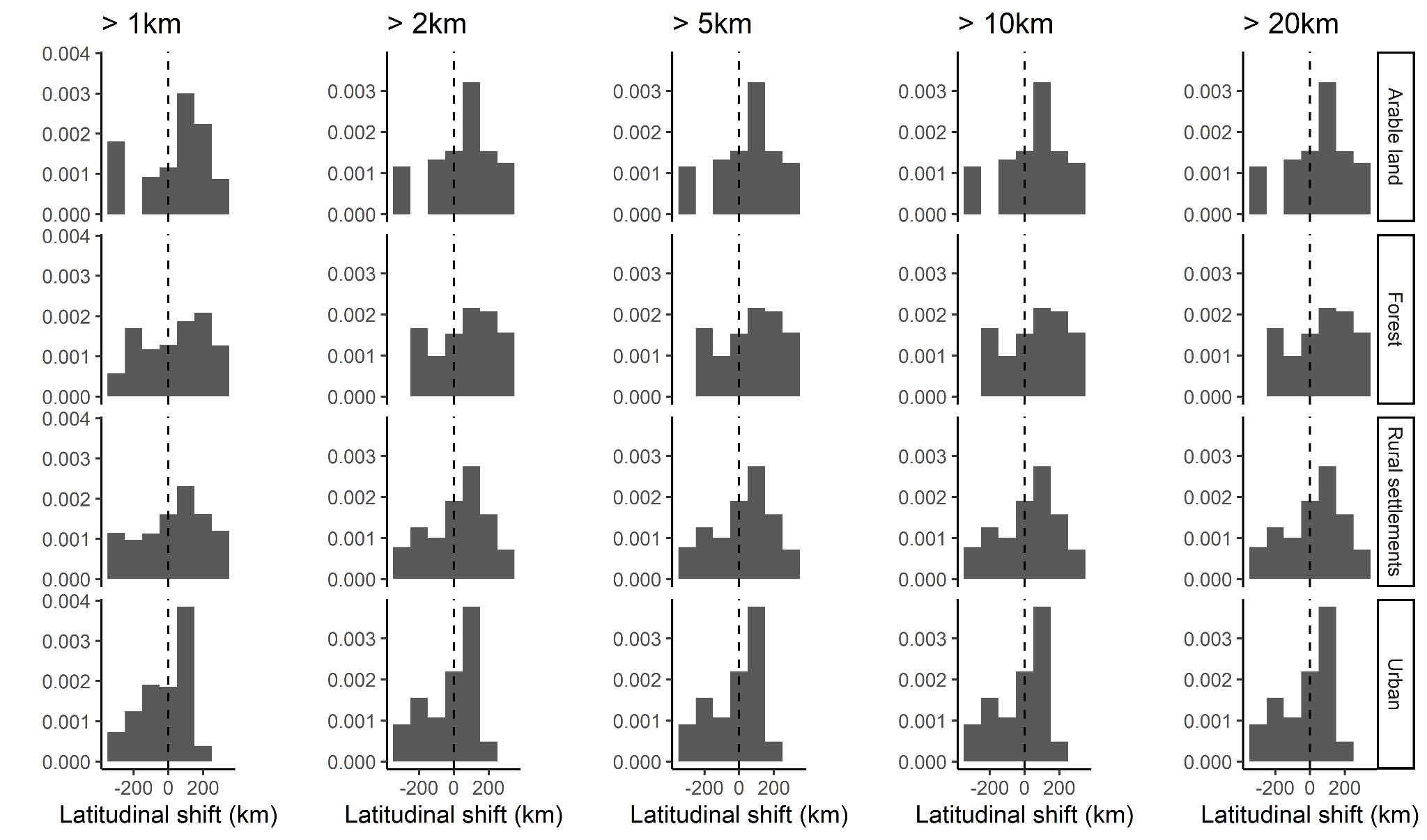


**Fig. S14.** Sensitivity analysis for the effect of grid survey effort. We summarized the distributions of the northward shift with different route length thresholds (1 km/2 km/5 km/10 km/20 km) for finding the optimal threshold to exclude grids for our analyses.

**Sensitivity Analyses:**

1. **Eastward and Northward shift directions without model weights**

To understand the effect of model weights we used in the main models presented in the paper (model weights = shift distance, with higher weighing for longer shift distances), we ran the same set of models for eastward and northward shift directions. For eastward shift directions there was only one model slightly different (m3), while for northward shift directions, all five models differed from the models including weights. This implies that mainly the longer shifts showed stronger relationships in their directionality along the latitudinal axis with our predictor variables.

**Table S4.** Model outputs (AIC values, estimates, standard errors SE, t and p values) for all five models fitted without shift distance specified as model weights (which were included in the models presented in the main ms) **for eastward and northward shift directions**. For each model, the sample size (N), AIC, AIC weight (w) and conditional *R^2^* are given. Significant and marginal effects (p<0.1) are depicted in bold. Continuous predictors were standardized prior to modeling. For the factor habitat, arable land was used as reference level in all models.

| **Term** | **Beta*** | **SE*** | **t-value** | **p-value** |
| --- | --- | --- | --- | --- |
| *m1: eastward ~ habitat, N = 234, AIC =477.162, AIC w = 0.173, R^2^ = 0.213* | | | | |
| Intercept | 0.074 | 0.102 | 0.725 | 0.469 |
| Forest | -0.085 | 0.122 | -0.700 | 0.485 |
| Rural settlement | -0.008 | 0.123 | -0.068 | 0.946 |
| Urban | -0.189 | 0.121 | -1.563 | 0.120 |
| Random: species (N = 81) | 0.301 |  |  |  |
| Random: residual | 0.596 |  |  |  |
| *m2: eastward ~ habitat + STI, N = 234, AIC = 478.041, AIC w = 0.111, R^2^ = 0.217* | | | | |
| Intercept | 0.077 | 0.102 | 0.751 | 0.454 |
| Forest | -0.097 | 0.122 | -0.793 | 0.429 |
| Rural settlement | -0.016 | 0.123 | -0.126 | 0.899 |
| Urban | -0.180 | 0.121 | -1.493 | 0.137 |
| STI | -0.054 | 0.051 | -1.060 | 0.292 |
| Random: species (N = 81) | 0.299 |  |  |  |
| Random: residual | 0.595 |  |  |  |
| *m3: eastward ~ habitat * STI, N = 234, AIC = 481.104, AIC w = 0.024, R^2^ = 0.239* | | | | |
| Intercept | 0.085 | 0.102 | 0.832 | 0.406 |
| Forest | -0.086 | 0.122 | -0.708 | 0.480 |
| Rural settlement | -0.033 | 0.122 | -0.274 | 0.784 |
| Urban | -0.192 | 0.121 | -1.590 | 0.114 |
| *STI | -0.139 | 0.119 | -1.171 | 0.243 |
| Forest:STI | 0.167 | 0.136 | 1.225 | 0.222 |
| Rural settlement:STI | -0.005 | 0.140 | -0.035 | 0.972 |
| Urban:STI | 0.103 | 0.134 | 0.767 | 0.444 |
| Random: species (N = 81) | 0.307 |  |  |  |
| Random: residual | 0.588 |  |  |  |
| *m4: eastward ~ habitat + snow P2, N = 234, AIC = 474.931, AIC w = 0.527, R^2^ = 0.250* | | | | |
| Intercept | 0.015 | 0.105 | 0.146 | 0.884 |
| Forest | -0.016 | 0.125 | -0.127 | 0.899 |
| Rural settlement | 0.074 | 0.127 | 0.582 | 0.561 |
| Urban | -0.129 | 0.122 | -1.059 | 0.291 |
| **Snow P2** | **-0.104** | **0.050** | **-2.085** | **0.039** |
| Random: species (N = 81) | 0.314 |  |  |  |
| Random: residual | 0.585 |  |  |  |
| *m5: eastward ~ habitat * snow P2, N = 234, AIC = 477.253, AIC w = 0.165, R^2^ = 0.270* | | | | |
| Intercept | 0.022 | 0.145 | 0.153 | 0.879 |
| Forest | -0.012 | 0.157 | -0.078 | 0.938 |
| Rural settlement | 0.040 | 0.159 | 0.252 | 0.801 |
| Urban | -0.140 | 0.157 | -0.891 | 0.374 |
| Snow P2 | -0.083 | 0.196 | -0.424 | 0.672 |
| Forest:snow P2 | -0.087 | 0.203 | -0.426 | 0.671 |
| Rural settlement:snow P2 | 0.077 | 0.204 | 0.380 | 0.704 |
| Urban:snow P2 | -0.082 | 0.212 | -0.388 | 0.699 |
| Random: species (N = 81) | 0.321 |  |  |  |
| Random: residual | 0.578 |  |  |  |
|  | | | | |
| *m1: northward ~ habitat, N = 234, AIC = 519.520, AIC w = 0.342, R^2^ = 0.228* | | | | |
| Intercept | 0.198 | 0.112 | 1.763 | **0.079** |
| *Forest | -0.172 | 0.133 | -1.294 | 0.197 |
| *Rural settlement | -0.093 | 0.133 | -0.694 | 0.488 |
| Urban | -0.130 | 0.131 | -0.992 | 0.322 |
| Random: species (N = 81) | 0.346 |  |  |  |
| Random: residual | 0.647 |  |  |  |
| *m2: northward ~ habitat + STI, N = 234, AIC = 520.985, AIC w = 0.164, R^2^ = 0.229* | | | | |
| Intercept | 0.200 | 0.112 | 1.781 | **0.076** |
| *Forest | -0.180 | 0.133 | -1.354 | 0.177 |
| *Rural settlement | -0.098 | 0.133 | -0.732 | 0.465 |
| Urban | -0.124 | 0.131 | -0.943 | 0.347 |
| STI | -0.041 | 0.057 | -0.732 | 0.466 |
| Random: species (N = 81) | 0.345 |  |  |  |
| Random: residual | 0.646 |  |  |  |
| *m3: northward ~ habitat * STI, N = 234, AIC = 525.469, AIC w = 0.017, R^2^ = 0.233* | | | | |
| Intercept | 0.193 | 0.112 | 1.718 | **0.087** |
| *Forest | -0.185 | 0.134 | -1.385 | 0.168 |
| *Rural settlement | -0.103 | 0.134 | -0.768 | 0.443 |
| Urban | -0.137 | 0.133 | -1.033 | 0.303 |
| STI | -0.033 | 0.131 | -0.254 | 0.800 |
| Forest:STI | -0.051 | 0.149 | -0.339 | 0.735 |
| Rural settlement:STI | -0.078 | 0.154 | -0.507 | 0.613 |
| Urban:STI | 0.057 | 0.147 | 0.391 | 0.696 |
| Random: species (N = 81) | 0.343 |  |  |  |
| Random: residual | 0.644 |  |  |  |
| *m4: northward ~ habitat + snow P2, N = 234, AIC = 519.571, AIC w = 0.334, R^2^ = 0.233* | | | | |
| Intercept | 0.241 | 0.116 | 2.080 | **0.039** |
| *Forest | -0.223 | 0.137 | -1.626 | 0.106 |
| *Rural settlement | -0.151 | 0.139 | -1.085 | 0.279 |
| *Urban | -0.173 | 0.134 | -1.288 | 0.199 |
| *Snow P2 | 0.076 | 0.055 | 1.400 | 0.164 |
| Random: species (N = 81) | 0.342 |  |  |  |
| Random: residual | 0.645 |  |  |  |
| *m5: northward ~ habitat * snow P2, N = 234, AIC = 521.274, AIC w = 0.142, R^2^ = 0.244* | | | | |
| Intercept | 0.139 | 0.159 | 0.875 | 0.382 |
| Forest | -0.128 | 0.174 | -0.737 | 0.462 |
| Rural settlement | -0.077 | 0.176 | -0.440 | 0.660 |
| Urban | -0.083 | 0.173 | -0.482 | 0.630 |
| Snow P2 | -0.098 | 0.216 | -0.452 | 0.652 |
| *Forest:snow P2 | 0.183 | 0.224 | 0.817 | 0.415 |
| *Rural settlement:snow P2 | 0.264 | 0.225 | 1.176 | 0.241 |
| Urban:snow P2 | 0.015 | 0.234 | 0.063 | 0.950 |
| Random: species (N = 81) | 0.339 |  |  |  |
| Random: residual | 0.639 |  |  |  |

**For random effects (species), the variance and standard deviation are shown.*

### Table S5. Summary table of habitat specific average latitudinal distances, overall distances and northern margin distances shifted across all species (all in [km]), median snow depth per period [cm] and number of species per habitat type.

| **Habitat** | **Lat. distance** | **Distance** | **Northern margin distance** | **Snow depth P1 (cm)** | **Snow depth P2 (cm)** | **Nr. of species** |
| --- | --- | --- | --- | --- | --- | --- |
| Arable land | 27.80 | 120.42 | 36.52 | 11.30 | 5.54 | 41 |
| Forest | 12.15 | 92.87 | 35.09 | 16.83 | 8.55 | 64 |
| Rural settlement | 15.82 | 105.84 | 45.74 | 16.25 | 8.29 | 61 |
| Urban | 2.28 | 99.00 | 44.39 | 15.61 | 6.29 | 68 |


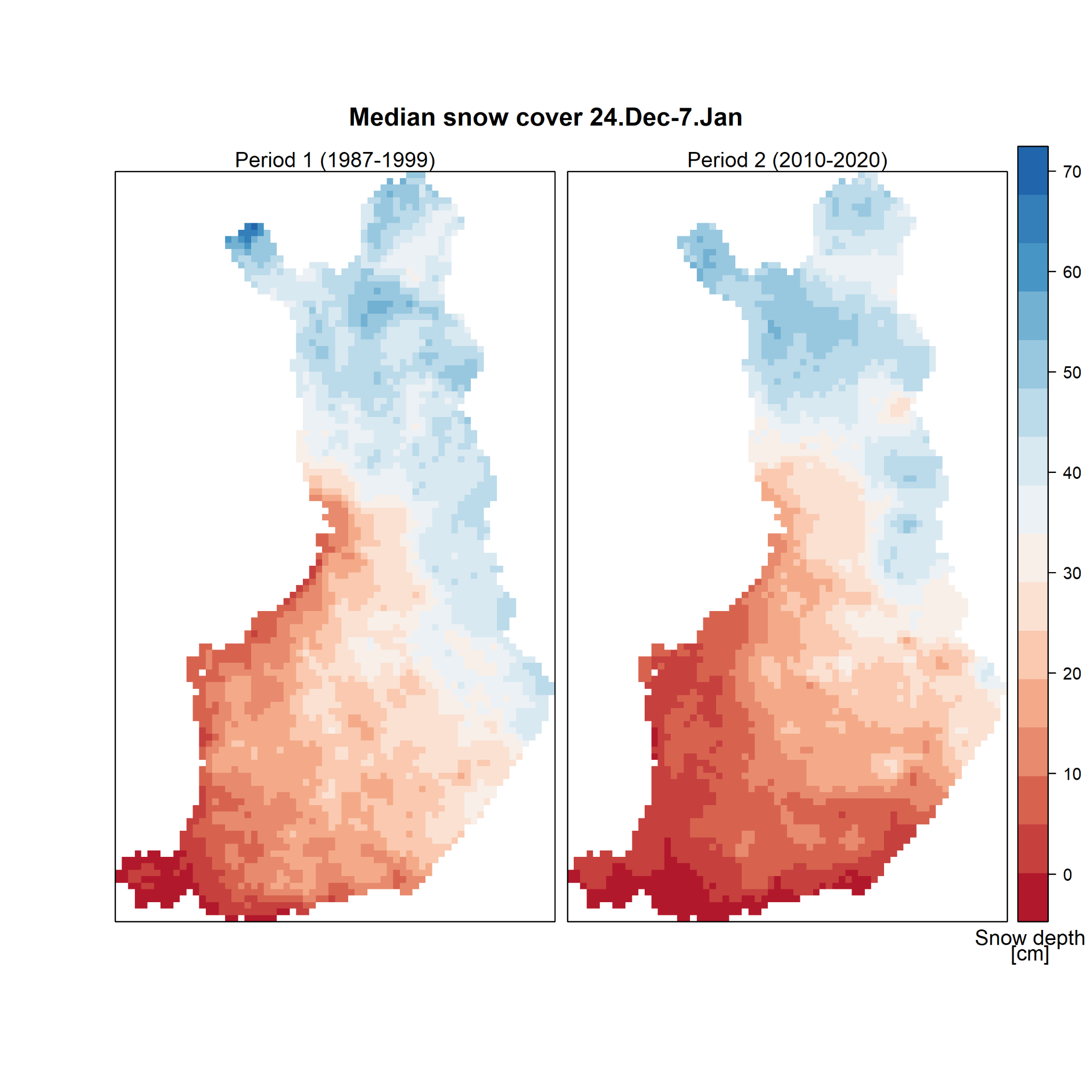


**Fig. S15.** Median snow depth (cm) per winter, averaged across all years for period one (left) and two (right).


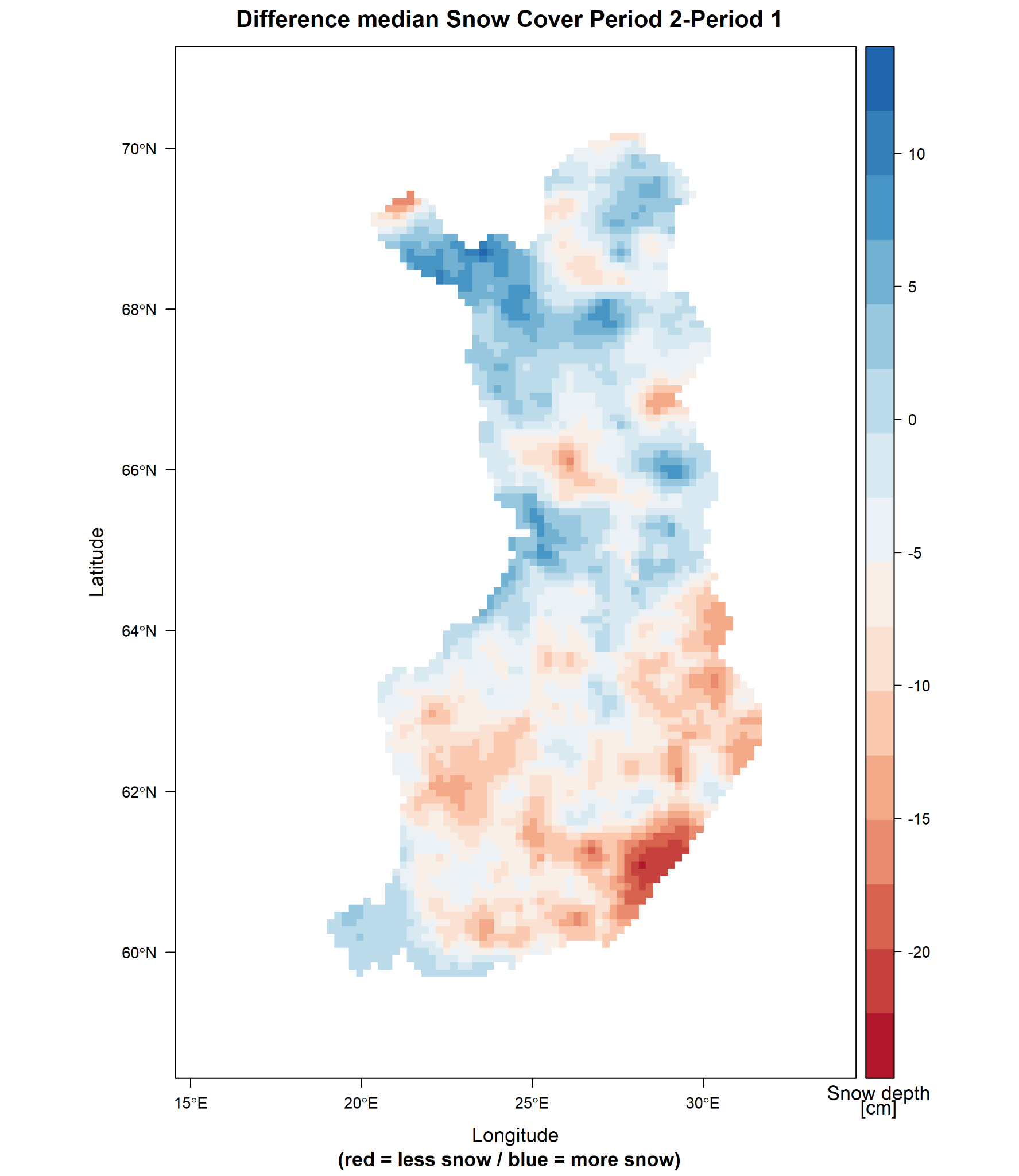


**Fig. S16.** Change in median snow depth (cm) from period one to period two.

### Table S6. Raw data with habitat-specific shift distances of northern range margins (NM), overall distance and latitudinal distance (all in km). Also given are, which is the main (=1) habitat per species, the shift direction (in degrees), the habitat specialization (relative density), the species temperature index STI and whether the observation was included in the NM analysis (=1) or not (=0).

| **Species** | **Habitat** | **Main** | **Shift NM** | **Shift distance** | **Shift dir.** | **Shift lat dist** | **Rel. Density** | **STI** | **NM analysis** |
| --- | --- | --- | --- | --- | --- | --- | --- | --- | --- |
| *Acanthis flammea* | arable land | 0 | 39.29 | 70.21 | 321.77 | 62.63 | 0.51 | -7.44 | 0 |
| *Acanthis flammea* | forest | 0 | -2.67 | 81.76 | 258.38 | -79.59 | 0.73 | -7.44 | 1 |
| *Acanthis flammea* | rural settlements | 0 | 58.21 | 33.69 | 342.59 | 12.83 | 1.34 | -7.44 | 1 |
| *Acanthis flammea* | urban | 1 | 65.83 | 75.38 | 237.75 | -40.22 | 1.47 | -7.44 | 1 |
| *Acanthis hornemanni* | arable land | 0 | -180.00 | 126.79 | 192.30 | -120.71 | 0.62 | -7.44 | 0 |
| *Acanthis hornemanni* | forest | 0 | -175.00 | 55.30 | 193.22 | -24.41 | 0.33 | -7.44 | 1 |
| *Acanthis hornemanni* | rural settlements | 0 | -73.33 | 70.62 | 102.97 | -70.54 | 1.49 | -7.44 | 1 |
| *Acanthis hornemanni* | urban | 1 | 21.11 | 119.05 | 183.21 | -118.86 | 1.96 | -7.44 | 1 |
| *Accipiter gentilis* | arable land | 0 | 115.00 | 176.60 | 1.43 | 158.99 | 0.52 | -6.25 | 0 |
| *Accipiter gentilis* | forest | 0 | 148.44 | 37.12 | 347.70 | 29.67 | 0.63 | -6.25 | 0 |
| *Accipiter gentilis* | rural settlements | 0 | -6.67 | 97.06 | 68.45 | 93.12 | 1.01 | -6.25 | 1 |
| *Accipiter gentilis* | urban | 1 | -0.81 | 65.96 | 241.07 | -31.90 | 1.89 | -6.25 | 1 |
| *Accipiter nisus* | arable land | 0 | -67.22 | 97.18 | 339.80 | 86.41 | 0.29 | 2.79 | 0 |
| *Accipiter nisus* | forest | 0 | 18.33 | 111.88 | 196.82 | -84.33 | 0.32 | 2.79 | 0 |
| *Accipiter nisus* | rural settlements | 0 | -67.73 | 35.35 | 203.86 | -33.68 | 1.84 | 2.79 | 0 |
| *Accipiter nisus* | urban | 1 | 1.67 | 43.29 | 222.10 | -32.12 | 1.89 | 2.79 | 0 |
| *Aegithalos caudatus* | arable land | 0 | 193.33 | 250.13 | 330.14 | 75.25 | 0.06 | -5.07 | 0 |
| *Aegithalos caudatus* | forest | 1 | 262.98 | 41.44 | 245.05 | -15.52 | 1.49 | -5.07 | 0 |
| *Aegithalos caudatus* | rural settlements | 0 | 65.33 | 7.47 | 83.27 | 6.70 | 1.02 | -5.07 | 0 |
| *Aegithalos caudatus* | urban | 0 | 218.33 | 66.04 | 57.75 | 31.38 | 0.68 | -5.07 | 0 |
| *Anas platyrhynchos* | arable land | 0 | 9.83 | 23.89 | 162.59 | -23.50 | 0.02 | 6.09 | 0 |
| *Anas platyrhynchos* | forest | 0 | 369.67 | 15.72 | 42.05 | 6.35 | 0.08 | 6.09 | 0 |
| *Anas platyrhynchos* | rural settlements | 0 | -19.05 | 13.57 | 46.01 | 11.38 | 0.10 | 6.09 | 0 |
| *Anas platyrhynchos* | urban | 1 | 52.30 | 33.81 | 241.63 | -1.38 | 3.84 | 6.09 | 0 |
| *Aythya fuligula* | urban | 1 | -305.56 | 126.03 | 267.66 | -59.44 | 2.98 | 12.06 | 0 |
| *Bombycilla garrulus* | arable land | 0 | -36.11 | 107.64 | 262.23 | -36.16 | 0.06 | -6.21 | 0 |
| *Bombycilla garrulus* | forest | 0 | 135.69 | 67.18 | 277.77 | 17.28 | 0.12 | -6.21 | 0 |
| *Bombycilla garrulus* | rural settlements | 0 | 157.78 | 107.44 | 289.10 | 61.11 | 0.81 | -6.21 | 0 |
| *Bombycilla garrulus* | urban | 1 | -2.80 | 118.15 | 302.25 | 112.38 | 3.18 | -6.21 | 1 |
| *Branta canadensis* | arable land | 0 | 3.33 | 149.87 | 109.10 | -22.00 | 1.33 | -0.11 | 0 |
| *Branta canadensis* | urban | 1 | -146.33 | 338.86 | 122.25 | -318.14 | 2.85 | -0.11 | 0 |
| *Bubo bubo* | forest | 1 | 10.83 | 5.68 | 358.57 | 4.65 | 1.77 | -6.9 | 0 |
| *Bucephala clangula* | urban | 1 | 111.11 | 85.93 | 176.79 | -19.28 | 3.36 | -0.27 | 0 |
| *Carduelis carduelis* | arable land | 0 | 116.11 | 112.92 | 293.84 | 14.37 | 0.83 | 2.79 | 0 |
| *Carduelis carduelis* | forest | 0 | 258.33 | 183.89 | 156.14 | -21.15 | 0.22 | 2.79 | 0 |
| *Carduelis carduelis* | rural settlements | 0 | 44.00 | 30.84 | 291.55 | 8.06 | 1.31 | 2.79 | 0 |
| *Carduelis carduelis* | urban | 1 | 96.67 | 60.35 | 356.79 | 20.60 | 2.17 | 2.79 | 0 |
| *Certhia familiaris* | forest | 1 | 70.37 | 22.92 | 172.14 | -22.92 | 1.84 | -6.89 | 0 |
| *Certhia familiaris* | rural settlements | 0 | 18.71 | 38.64 | 77.74 | 31.38 | 0.65 | -6.89 | 0 |
| *Certhia familiaris* | urban | 0 | 64.81 | 49.31 | 118.93 | -48.81 | 0.42 | -6.89 | 0 |
| *Chloris chloris* | arable land | 0 | -91.11 | 25.71 | 111.55 | -0.31 | 0.12 | -0.96 | 0 |
| *Chloris chloris* | forest | 0 | 211.67 | 327.44 | 51.31 | 318.17 | 0.05 | -0.96 | 0 |
| *Chloris chloris* | rural settlements | 0 | 41.11 | 117.08 | 23.86 | 108.67 | 2.10 | -0.96 | 1 |
| *Chloris chloris* | urban | 1 | 36.67 | 59.81 | 3.21 | 51.17 | 2.22 | -0.96 | 1 |
| *Cinclus cinclus* | forest | 0 | 113.33 | 219.01 | 15.32 | 207.53 | 0.70 | -5.63 | 0 |
| *Cinclus cinclus* | rural settlements | 1 | 301.67 | 233.36 | 276.73 | 232.95 | 2.01 | -5.63 | 0 |
| *Cinclus cinclus* | urban | 0 | 90.00 | 97.63 | 61.07 | 67.30 | 1.18 | -5.63 | 0 |
| *Clangula hyemalis* | forest | 0 | 120.00 | 188.55 | 28.97 | 180.97 | 0.28 | -1.65 | 0 |
| *Clangula hyemalis* | urban | 1 | 15.00 | 112.16 | 42.10 | 29.51 | 3.57 | -1.65 | 0 |
| *Coccothraustes coccothraustes* | rural settlements | 0 | -523.33 | 271.05 | 187.86 | -252.48 | 1.45 | 0.11 | 0 |
| *Coccothraustes coccothraustes* | urban | 1 | -66.67 | 419.15 | 241.86 | -181.62 | 2.49 | 0.11 | 0 |
| *Columba livia* | arable land | 0 | -23.33 | 131.56 | 181.43 | -121.79 | 0.02 | 7.25 | 0 |
| *Columba livia* | forest | 0 | 206.67 | 296.42 | 71.02 | 258.67 | 0.01 | 7.25 | 0 |
| *Columba livia* | rural settlements | 0 | 133.33 | 93.49 | 109.96 | -47.88 | 0.37 | 7.25 | 0 |
| *Columba livia* | urban | 1 | -0.52 | 51.88 | 298.93 | 51.22 | 3.73 | 7.25 | 1 |
| *Corvus corax* | arable land | 0 | 126.67 | 29.98 | 24.97 | 15.81 | 0.68 | -5.41 | 0 |
| *Corvus corax* | forest | 0 | 33.89 | 78.15 | 153.55 | -77.59 | 0.97 | -5.41 | 1 |
| *Corvus corax* | rural settlements | 0 | 28.33 | 278.29 | 171.81 | -278.27 | 0.73 | -5.41 | 1 |
| *Corvus corax* | urban | 1 | 42.50 | 163.82 | 137.90 | -156.31 | 1.44 | -5.41 | 1 |
| *Corvus corone* | arable land | 0 | 115.33 | 47.21 | 96.73 | -16.34 | 0.74 | -4.93 | 0 |
| *Corvus corone* | forest | 0 | 86.67 | 9.82 | 335.03 | 2.25 | 0.11 | -4.93 | 1 |
| *Corvus corone* | rural settlements | 0 | 71.44 | 97.36 | 7.86 | 71.74 | 1.10 | -4.93 | 1 |
| *Corvus corone* | urban | 1 | 38.48 | 26.30 | 197.98 | -8.61 | 2.57 | -4.93 | 1 |
| *Corvus frugilegus* | urban | 1 | -6.67 | 23.62 | 118.37 | -8.67 | 2.76 | -0.99 | 0 |
| *Corvus monedula* | arable land | 0 | 78.52 | 41.11 | 28.82 | 13.45 | 0.63 | -0.91 | 0 |
| *Corvus monedula* | forest | 0 | -36.67 | 31.98 | 39.51 | 31.65 | 0.02 | -0.91 | 0 |
| *Corvus monedula* | rural settlements | 0 | 146.98 | 144.41 | 26.45 | 74.66 | 1.17 | -0.91 | 0 |
| *Corvus monedula* | urban | 1 | 197.19 | 45.93 | 61.63 | 5.39 | 2.73 | -0.91 | 0 |
| *Cyanistes caeruleus* | arable land | 0 | 230.00 | 108.02 | 45.58 | 107.21 | 0.03 | -2.94 | 0 |
| *Cyanistes caeruleus* | forest | 0 | 243.33 | 40.84 | 6.93 | 9.46 | 0.38 | -2.94 | 0 |
| *Cyanistes caeruleus* | rural settlements | 1 | 199.51 | 42.41 | 25.05 | 38.19 | 2.35 | -2.94 | 1 |
| *Cyanistes caeruleus* | urban | 0 | 64.74 | 34.28 | 87.66 | 23.81 | 1.51 | -2.94 | 1 |
| *Cygnus cygnus* | arable land | 1 | 105.71 | 48.51 | 9.21 | 26.82 | 4.69 | -1.19 | 0 |
| *Cygnus cygnus* | rural settlements | 0 | 90.71 | 144.71 | 9.23 | 4.63 | 0.98 | -1.19 | 0 |
| *Cygnus cygnus* | urban | 0 | 80.00 | 221.06 | 317.90 | 46.92 | 0.49 | -1.19 | 0 |
| *Cygnus olor* | forest | 0 | -404.33 | 264.11 | 262.20 | -263.19 | 0.38 | 0.85 | 0 |
| *Cygnus olor* | rural settlements | 0 | -11.67 | 129.07 | 206.45 | -9.38 | 0.62 | 0.85 | 0 |
| *Cygnus olor* | urban | 1 | -13.33 | 64.40 | 61.86 | 58.90 | 2.89 | 0.85 | 0 |
| *Dendrocopos major* | arable land | 0 | 36.67 | 121.22 | 204.97 | -0.91 | 0.04 | -5.79 | 0 |
| *Dendrocopos major* | forest | 0 | 3.33 | 48.02 | 268.93 | -42.14 | 1.05 | -5.79 | 1 |
| *Dendrocopos major* | rural settlements | 1 | 19.52 | 23.48 | 205.05 | -22.55 | 1.76 | -5.79 | 1 |
| *Dendrocopos major* | urban | 0 | 113.02 | 57.81 | 200.14 | -57.27 | 0.85 | -5.79 | 0 |
| *Dendrocopos minor* | forest | 0 | -115.00 | 82.32 | 183.76 | -73.62 | 1.14 | -6.47 | 0 |
| *Dendrocopos minor* | rural settlements | 1 | 81.67 | 52.01 | 148.83 | -7.10 | 1.59 | -6.47 | 0 |
| *Dendrocopos minor* | urban | 0 | -48.33 | 99.64 | 257.03 | -89.21 | 0.80 | -6.47 | 0 |
| *Dryocopus martius* | forest | 1 | 52.04 | 27.78 | 219.36 | -23.08 | 1.91 | -7.59 | 0 |
| *Dryocopus martius* | rural settlements | 0 | 6.94 | 51.15 | 52.08 | 49.76 | 0.67 | -7.59 | 0 |
| *Dryocopus martius* | urban | 0 | 100.00 | 6.80 | 92.34 | -6.16 | 0.26 | -7.59 | 0 |
| *Emberiza citrinella* | arable land | 0 | 13.20 | 26.78 | 133.99 | -0.23 | 0.96 | -5.2 | 0 |
| *Emberiza citrinella* | forest | 0 | -34.38 | 17.54 | 208.43 | -11.07 | 0.09 | -5.2 | 0 |
| *Emberiza citrinella* | rural settlements | 1 | 148.83 | 46.98 | 31.72 | 35.18 | 2.94 | -5.2 | 0 |
| *Emberiza citrinella* | urban | 0 | -98.19 | 39.98 | 298.37 | 39.46 | 1.02 | -5.2 | 1 |
| *Erithacus rubecula* | forest | 0 | 118.43 | 18.73 | 331.18 | 9.48 | 1.00 | 3.81 | 0 |
| *Erithacus rubecula* | rural settlements | 0 | -6.67 | 157.47 | 189.23 | -47.43 | 1.24 | 3.81 | 0 |
| *Erithacus rubecula* | urban | 1 | 32.50 | 32.18 | 250.04 | -19.78 | 1.35 | 3.81 | 0 |
| *Fringilla coelebs* | arable land | 0 | 6.67 | 326.54 | 33.11 | 104.06 | 0.44 | 1.51 | 0 |
| *Fringilla coelebs* | forest | 0 | -11.11 | 71.31 | 154.95 | -8.61 | 0.09 | 1.51 | 0 |
| *Fringilla coelebs* | rural settlements | 1 | -138.33 | 60.21 | 133.58 | -0.49 | 2.23 | 1.51 | 0 |
| *Fringilla coelebs* | urban | 0 | -98.72 | 24.37 | 188.19 | -20.73 | 1.88 | 1.51 | 0 |
| *Fringilla montifringilla* | arable land | 0 | 103.33 | 81.34 | 11.23 | 24.46 | 0.53 | 1.43 | 0 |
| *Fringilla montifringilla* | forest | 0 | 251.67 | 40.73 | 314.42 | 39.62 | 0.06 | 1.43 | 0 |
| *Fringilla montifringilla* | rural settlements | 0 | -177.78 | 50.04 | 232.08 | -49.52 | 1.76 | 1.43 | 0 |
| *Fringilla montifringilla* | urban | 1 | -108.33 | 88.93 | 17.98 | 69.86 | 2.24 | 1.43 | 0 |
| *Fulica atra* | urban | 1 | -173.33 | 58.83 | 272.34 | 55.21 | 2.43 | 7.06 | 0 |
| *Garrulus glandarius* | arable land | 0 | 93.50 | 61.00 | 78.38 | 60.56 | 0.18 | -4.93 | 0 |
| *Garrulus glandarius* | forest | 0 | 21.67 | 17.20 | 170.77 | -9.69 | 0.85 | -4.93 | 1 |
| *Garrulus glandarius* | rural settlements | 1 | 52.78 | 22.72 | 105.26 | -22.71 | 2.55 | -4.93 | 1 |
| *Garrulus glandarius* | urban | 0 | -2.08 | 18.92 | 298.14 | 16.41 | 0.47 | -4.93 | 0 |
| *Glaucidium passerinum* | forest | 0 | 186.43 | 38.76 | 127.92 | -9.46 | 1.03 | -9.86 | 0 |
| *Glaucidium passerinum* | rural settlements | 1 | -64.17 | 74.88 | 211.72 | -26.54 | 2.04 | -9.86 | 0 |
| *Glaucidium passerinum* | urban | 0 | 388.33 | 193.16 | 20.14 | 78.09 | 0.53 | -9.86 | 0 |
| *Lagopus lagopus* | forest | 1 | 0.00 | 102.56 | 350.79 | 102.56 | 2.03 | -10.35 | 1 |
| *Lagopus lagopus* | rural settlements | 0 | 215.56 | 337.84 | 313.99 | 295.95 | 0.40 | -10.35 | 0 |
| *Lanius excubitor* | arable land | 1 | 195.00 | 35.97 | 300.50 | 35.97 | 4.07 | 6.59 | 0 |
| *Lanius excubitor* | forest | 0 | -59.44 | 25.38 | 222.05 | -3.55 | 0.40 | 6.59 | 0 |
| *Lanius excubitor* | rural settlements | 0 | 40.00 | 58.06 | 282.26 | 42.00 | 0.90 | 6.59 | 0 |
| *Lanius excubitor* | urban | 0 | 327.78 | 202.36 | 77.03 | 102.72 | 0.38 | 6.59 | 0 |
| *Larus argentatus* | arable land | 0 | 170.00 | 115.87 | 329.59 | 98.43 | 0.12 | 0.66 | 0 |
| *Larus argentatus* | forest | 0 | 226.67 | 225.60 | 326.89 | 164.76 | 0.02 | 0.66 | 0 |
| *Larus argentatus* | rural settlements | 0 | 52.08 | 180.70 | 336.14 | 154.85 | 0.15 | 0.66 | 0 |
| *Larus argentatus* | urban | 1 | 79.29 | 199.55 | 70.04 | 172.10 | 3.82 | 0.66 | 0 |
| *Larus canus* | rural settlements | 0 | 206.33 | 189.80 | 352.14 | 188.60 | 0.06 | 1.8 | 0 |
| *Larus canus* | urban | 1 | 163.33 | 77.06 | 8.19 | 76.88 | 3.76 | 1.8 | 0 |
| *Larus marinus* | rural settlements | 0 | 41.67 | 78.30 | 218.23 | -2.16 | 0.61 | 1.13 | 0 |
| *Larus marinus* | urban | 1 | 85.37 | 169.85 | 342.02 | 146.58 | 3.30 | 1.13 | 0 |
| *Linaria cannabina* | arable land | 1 | 115.28 | 64.77 | 356.08 | 55.62 | 2.84 | 1.93 | 0 |
| *Linaria cannabina* | rural settlements | 0 | -13.00 | 156.97 | 38.23 | 12.99 | 1.15 | 1.93 | 0 |
| *Linaria cannabina* | urban | 0 | -33.89 | 78.76 | 31.17 | 38.79 | 1.43 | 1.93 | 0 |
| *Lophophanes cristatus* | forest | 1 | -69.68 | 35.04 | 348.77 | 25.07 | 1.81 | -5.9 | 0 |
| *Lophophanes cristatus* | rural settlements | 0 | 2.67 | 10.85 | 20.20 | 10.82 | 0.99 | -5.9 | 0 |
| *Lophophanes cristatus* | urban | 0 | -92.78 | 52.55 | 118.14 | -27.53 | 0.19 | -5.9 | 0 |
| *Loxia curvirostra* | arable land | 0 | -21.11 | 281.36 | 13.22 | 280.74 | 0.05 | -7.53 | 0 |
| *Loxia curvirostra* | forest | 1 | -60.00 | 131.87 | 281.62 | 129.03 | 1.94 | -7.53 | 1 |
| *Loxia curvirostra* | rural settlements | 0 | -61.33 | 148.07 | 333.55 | 147.80 | 0.53 | -7.53 | 1 |
| *Loxia curvirostra* | urban | 0 | 22.22 | 136.41 | 339.86 | 133.28 | 0.32 | -7.53 | 1 |
| *Loxia leucoptera* | forest | 1 | 51.67 | 172.21 | 82.70 | 165.20 | 2.10 | -13.37 | 1 |
| *Loxia pytyopsittacus* | forest | 1 | -76.11 | 198.79 | 231.31 | -162.23 | 1.86 | -10.67 | 0 |
| *Loxia pytyopsittacus* | rural settlements | 0 | 10.00 | 325.68 | 200.20 | -246.13 | 0.44 | -10.67 | 0 |
| *Loxia pytyopsittacus* | urban | 0 | -83.89 | 91.06 | 282.97 | 12.31 | 0.54 | -10.67 | 0 |
| *Lyrurus tetrix* | arable land | 1 | 52.56 | 29.24 | 208.82 | -18.25 | 2.11 | -9.96 | 0 |
| *Lyrurus tetrix* | forest | 0 | 73.41 | 77.22 | 7.52 | 59.08 | 1.54 | -9.96 | 1 |
| *Lyrurus tetrix* | rural settlements | 0 | 109.44 | 101.42 | 115.68 | -100.98 | 0.33 | -9.96 | 0 |
| *Mergus merganser* | urban | 1 | -104.44 | 118.78 | 46.42 | 118.74 | 3.09 | 0.33 | 0 |
| *Mergus serrator* | urban | 1 | -13.33 | 69.32 | 211.17 | -62.84 | 3.99 | 0.88 | 0 |
| *Nucifraga caryocatactes* | forest | 0 | 223.10 | 258.13 | 73.46 | 230.55 | 1.06 | -9.2 | 0 |
| *Nucifraga caryocatactes* | rural settlements | 1 | 15.92 | 34.33 | 29.86 | 26.35 | 1.73 | -9.2 | 0 |
| *Nucifraga caryocatactes* | urban | 0 | 198.67 | 97.56 | 289.96 | 85.48 | 0.86 | -9.2 | 0 |
| *Panurus biarmicus* | forest | 1 | 61.11 | 35.86 | 67.61 | 26.54 | 2.08 | -1.73 | 0 |
| *Parus major* | arable land | 0 | -6.67 | 26.62 | 16.82 | 24.14 | 0.03 | -4.76 | 0 |
| *Parus major* | forest | 0 | 56.94 | 16.85 | 148.28 | -5.93 | 0.28 | -4.76 | 1 |
| *Parus major* | rural settlements | 1 | 64.87 | 14.83 | 66.16 | 10.09 | 2.14 | -4.76 | 1 |
| *Parus major* | urban | 0 | 37.17 | 42.42 | 74.74 | 29.69 | 1.86 | -4.76 | 1 |
| *Passer domesticus* | arable land | 0 | -297.04 | 259.50 | 225.58 | -258.86 | 0.02 | -2.61 | 0 |
| *Passer domesticus* | forest | 0 | -370.00 | 210.71 | 141.77 | -210.71 | 0.00 | -2.61 | 0 |
| *Passer domesticus* | rural settlements | 0 | 71.67 | 131.24 | 59.50 | 125.20 | 1.52 | -2.61 | 1 |
| *Passer domesticus* | urban | 1 | 66.86 | 14.63 | 351.81 | 14.44 | 2.82 | -2.61 | 1 |
| *Passer montanus* | arable land | 0 | 413.75 | 256.94 | 65.05 | 136.70 | 0.03 | -3.96 | 0 |
| *Passer montanus* | rural settlements | 1 | 376.67 | 94.08 | 334.95 | 90.85 | 2.25 | -3.96 | 0 |
| *Passer montanus* | urban | 0 | 283.17 | 113.25 | 328.83 | 94.87 | 2.23 | -3.96 | 0 |
| *Perdix perdix* | arable land | 1 | 75.24 | 27.53 | 82.20 | 14.12 | 2.68 | -5.37 | 0 |
| *Perdix perdix* | forest | 0 | -143.89 | 84.30 | 159.80 | -71.35 | 0.03 | -5.37 | 0 |
| *Perdix perdix* | rural settlements | 0 | -37.59 | 87.77 | 30.41 | 57.00 | 2.52 | -5.37 | 0 |
| *Periparus ater* | forest | 0 | -100.98 | 57.82 | 150.14 | -1.34 | 1.08 | -5.03 | 0 |
| *Periparus ater* | rural settlements | 1 | 62.21 | 24.80 | 170.85 | -13.97 | 1.87 | -5.03 | 0 |
| *Periparus ater* | urban | 0 | 49.97 | 83.91 | 226.42 | -82.31 | 0.72 | -5.03 | 0 |
| *Perisoreus infaustus* | forest | 0 | 65.67 | 96.41 | 346.78 | 69.58 | 1.34 | -12.56 | 1 |
| *Perisoreus infaustus* | rural settlements | 1 | 149.33 | 91.12 | 3.92 | 85.17 | 2.04 | -12.56 | 1 |
| *Phalacrocorax carbo* | urban | 1 | 54.58 | 84.91 | 64.32 | 17.10 | 3.52 | 18.71 | 0 |
| *Phasianus colchicus* | arable land | 0 | 140.06 | 38.32 | 88.93 | 33.63 | 0.34 | -2.78 | 0 |
| *Phasianus colchicus* | forest | 0 | 3.49 | 62.54 | 343.18 | 51.36 | 0.06 | -2.78 | 0 |
| *Phasianus colchicus* | rural settlements | 1 | 56.80 | 34.61 | 30.34 | 30.44 | 2.40 | -2.78 | 0 |
| *Phasianus colchicus* | urban | 0 | 217.69 | 112.83 | 9.15 | 109.83 | 1.84 | -2.78 | 0 |
| *Pica pica* | arable land | 0 | 61.15 | 15.87 | 3.76 | 6.68 | 0.33 | -5.41 | 0 |
| *Pica pica* | forest | 0 | 50.00 | 29.88 | 2.67 | 18.42 | 0.13 | -5.41 | 1 |
| *Pica pica* | rural settlements | 0 | 76.33 | 54.61 | 60.49 | 47.96 | 1.99 | -5.41 | 1 |
| *Pica pica* | urban | 1 | 37.17 | 26.92 | 254.74 | -25.77 | 2.06 | -5.41 | 1 |
| *Picoides tridactylus* | forest | 1 | -222.22 | 249.37 | 113.84 | -244.36 | 2.06 | -10.52 | 1 |
| *Picus canus* | forest | 0 | 133.33 | 186.81 | 16.37 | 68.51 | 0.81 | -6.26 | 0 |
| *Picus canus* | rural settlements | 1 | 345.56 | 284.40 | 58.41 | 174.29 | 2.91 | -6.26 | 0 |
| *Picus canus* | urban | 0 | 161.11 | 288.20 | 17.41 | 121.56 | 0.35 | -6.26 | 0 |
| *Pinicola enucleator* | forest | 0 | 107.33 | 58.88 | 17.64 | 17.02 | 0.71 | -12.49 | 0 |
| *Pinicola enucleator* | rural settlements | 0 | 52.18 | 41.84 | 209.86 | -18.82 | 0.92 | -12.49 | 0 |
| *Pinicola enucleator* | urban | 1 | 14.15 | 65.93 | 244.32 | -8.95 | 2.10 | -12.49 | 1 |
| *Plectrophenax nivalis* | urban | 0 | 312.50 | 26.89 | 313.58 | 0.50 | 1.39 | -4.52 | 0 |
| *Poecile cinctus* | forest | 0 | -25.56 | 60.82 | 294.95 | 37.70 | 1.06 | -13.7 | 1 |
| *Poecile cinctus* | rural settlements | 1 | 54.82 | 47.41 | 12.30 | 26.76 | 2.61 | -13.7 | 1 |
| *Poecile montanus* | arable land | 0 | -20.83 | 184.18 | 329.66 | 183.68 | 0.03 | -7.51 | 0 |
| *Poecile montanus* | forest | 0 | -4.31 | 90.38 | 26.37 | 87.94 | 1.30 | -7.51 | 1 |
| *Poecile montanus* | rural settlements | 1 | 53.23 | 69.85 | 350.77 | 69.73 | 1.88 | -7.51 | 1 |
| *Poecile montanus* | urban | 0 | 32.92 | 148.82 | 70.90 | 148.50 | 0.34 | -7.51 | 1 |
| *Pyrrhula pyrrhula* | arable land | 0 | -79.47 | 113.91 | 299.51 | 95.69 | 0.11 | -4.58 | 0 |
| *Pyrrhula pyrrhula* | forest | 0 | 110.28 | 76.24 | 277.80 | 74.41 | 0.45 | -4.58 | 1 |
| *Pyrrhula pyrrhula* | rural settlements | 1 | 57.59 | 29.75 | 246.16 | -7.73 | 2.27 | -4.58 | 1 |
| *Pyrrhula pyrrhula* | urban | 0 | 36.65 | 49.38 | 189.15 | -38.18 | 1.42 | -4.58 | 1 |
| *Regulus regulus* | arable land | 0 | -25.00 | 286.93 | 39.36 | 178.63 | 0.01 | -4.23 | 0 |
| *Regulus regulus* | forest | 1 | -99.05 | 38.73 | 195.32 | -24.28 | 2.05 | -4.23 | 0 |
| *Regulus regulus* | rural settlements | 0 | -97.36 | 33.22 | 239.50 | -10.60 | 0.44 | -4.23 | 0 |
| *Regulus regulus* | urban | 0 | 133.89 | 73.71 | 197.41 | -64.82 | 0.22 | -4.23 | 0 |
| *Schoeniclus schoeniclus* | arable land | 0 | 110.00 | 203.89 | 28.43 | 203.89 | 0.87 | 2.12 | 0 |
| *Schoeniclus schoeniclus* | forest | 1 | 153.28 | 51.55 | 120.50 | -3.42 | 1.71 | 2.12 | 0 |
| *Schoeniclus schoeniclus* | urban | 0 | -6.67 | 178.68 | 162.02 | -132.68 | 0.35 | 2.12 | 0 |
| *Scolopax rusticola* | forest | 1 | 61.06 | 18.31 | 33.01 | 2.05 | 2.27 | 5.01 | 0 |
| *Sitta europaea* | forest | 0 | 271.33 | 129.46 | 208.97 | -98.28 | 0.63 | -5.01 | 0 |
| *Sitta europaea* | rural settlements | 1 | -244.44 | 245.12 | 210.41 | -203.94 | 1.91 | -5.01 | 0 |
| *Sitta europaea* | urban | 0 | -135.56 | 333.27 | 250.90 | -208.35 | 1.44 | -5.01 | 0 |
| *Spinus spinus* | arable land | 0 | -225.00 | 140.78 | 189.21 | -55.25 | 0.14 | 0.16 | 0 |
| *Spinus spinus* | forest | 0 | -166.67 | 53.86 | 251.02 | -30.11 | 0.81 | 0.16 | 0 |
| *Spinus spinus* | rural settlements | 0 | -56.67 | 145.21 | 183.92 | -62.36 | 1.07 | 0.16 | 0 |
| *Spinus spinus* | urban | 1 | -209.33 | 42.41 | 248.45 | -40.91 | 1.76 | 0.16 | 0 |
| *Streptopelia decaocto* | rural settlements | 0 | -104.00 | 146.92 | 210.34 | -70.26 | 0.92 | -2.83 | 0 |
| *Streptopelia decaocto* | urban | 1 | -14.09 | 63.14 | 159.86 | -55.24 | 3.29 | -2.83 | 0 |
| *Sturnus vulgaris* | arable land | 0 | 126.67 | 122.17 | 301.59 | 88.46 | 0.64 | 2.1 | 0 |
| *Sturnus vulgaris* | forest | 0 | -118.33 | 160.15 | 149.59 | -160.04 | 0.07 | 2.1 | 0 |
| *Sturnus vulgaris* | rural settlements | 0 | 135.00 | 125.51 | 240.49 | -125.20 | 0.78 | 2.1 | 0 |
| *Sturnus vulgaris* | urban | 1 | 12.22 | 71.57 | 285.26 | 23.28 | 2.95 | 2.1 | 0 |
| *Surnia ulula* | arable land | 1 | -276.67 | 327.49 | 102.26 | -324.65 | 2.56 | -12.25 | 0 |
| *Surnia ulula* | forest | 0 | -61.67 | 86.56 | 55.34 | 52.56 | 0.83 | -12.25 | 1 |
| *Sylvia atricapilla* | urban | 1 | 223.33 | 145.15 | 263.27 | -111.98 | 3.40 | 15.23 | 0 |
| *Tetrao urogallus* | forest | 1 | 115.83 | 111.70 | 74.86 | 97.08 | 2.28 | -9.78 | 0 |
| *Tetrastes bonasia* | forest | 1 | -129.30 | 60.20 | 271.07 | 60.15 | 2.26 | -9.64 | 1 |
| *Tetrastes bonasia* | rural settlements | 0 | 338.67 | 284.22 | 82.23 | 256.67 | 0.23 | -9.64 | 0 |
| *Troglodytes troglodytes* | forest | 1 | -36.11 | 19.44 | 219.51 | -8.38 | 1.83 | -0.58 | 0 |
| *Troglodytes troglodytes* | urban | 0 | 107.78 | 4.57 | 226.01 | -4.54 | 0.28 | -0.58 | 0 |
| *Turdus iliacus* | forest | 0 | -45.83 | 51.65 | 176.08 | -51.64 | 0.81 | 3.21 | 0 |
| *Turdus iliacus* | rural settlements | 0 | -10.00 | 170.18 | 238.41 | -94.41 | 0.76 | 3.21 | 0 |
| *Turdus iliacus* | urban | 1 | 203.33 | 191.15 | 257.74 | -24.29 | 1.95 | 3.21 | 0 |
| *Turdus merula* | arable land | 0 | -15.56 | 140.51 | 213.11 | -7.53 | 0.02 | 2.07 | 0 |
| *Turdus merula* | forest | 0 | 142.42 | 35.94 | 356.24 | 4.52 | 0.32 | 2.07 | 0 |
| *Turdus merula* | rural settlements | 0 | 31.68 | 20.21 | 307.92 | 16.90 | 1.49 | 2.07 | 0 |
| *Turdus merula* | urban | 1 | 104.58 | 19.75 | 295.68 | 19.58 | 2.30 | 2.07 | 0 |
| *Turdus pilaris* | arable land | 0 | 16.11 | 101.28 | 191.23 | -40.11 | 0.27 | -2.31 | 0 |
| *Turdus pilaris* | forest | 0 | -16.87 | 140.74 | 186.93 | -40.21 | 0.36 | -2.31 | 0 |
| *Turdus pilaris* | rural settlements | 0 | 216.19 | 132.52 | 328.28 | 79.45 | 0.94 | -2.31 | 0 |
| *Turdus pilaris* | urban | 1 | -11.90 | 134.17 | 350.85 | 38.19 | 2.54 | -2.31 | 1 |

**Literature**

Makowski D, Ben-Shachar MS, Waggoner P (2020) easystats/see: see 0.5.1. Zenodo. <https://doi.org/10.5281/ZENODO.3952153>

Lüdecke D, Ben-Shachar M, Patil I, Waggoner P, Makowski D (2021). “performance: An R Package for Assessment, Comparison and Testing of Statistical Models.” Journal of Open Source Software, 6(60), 3139. doi: 10.21105/joss.03139.
